# Methylome and transcriptome analyses of three different degrees of albinism in apple seedlings

**DOI:** 10.1186/s12864-022-08535-3

**Published:** 2022-04-19

**Authors:** Tingting Sun, Junke Zhang, Qiang Zhang, Xingliang Li, Minji Li, Yuzhang Yang, Jia Zhou, Qinping Wei, Beibei Zhou

**Affiliations:** Beijing Academy of Agriculture and Forestry Sciences, Beijing Academy of Forestry and Pomology Sciences, Beijing Engineering Research Center for Deciduous Fruit Trees, Key Laboratory of Biology and Genetic Improvement of Horticultural Crops, Ministry of Agriculture and Rural Affairs, Beijing, 100093 China

**Keywords:** Apple, Chlorophyll-deficient mutations, Methylation, Transcriptomes, Leaf colour

## Abstract

**Background:**

Leaf colour mutations are universally expressed at the seedling stage and are ideal materials for exploring the chlorophyll biosynthesis pathway, carotenoid metabolism and the flavonoid biosynthesis pathway in plants.

**Results:**

In this research, we analysed the different degrees of albinism in apple (*Malus domestica*) seedlings, including white-leaf mutants (WM), piebald leaf mutants (PM), light-green leaf mutants (LM) and normal leaves (NL) using bisulfite sequencing (BS-seq) and RNA sequencing (RNA-seq). There were 61,755, 79,824, and 74,899 differentially methylated regions (DMRs) and 7566, 3660, and 3546 differentially expressed genes (DEGs) identified in the WM/NL, PM/NL and LM/NL comparisons, respectively.

**Conclusion:**

The analysis of the methylome and transcriptome showed that 9 DMR-associated DEGs were involved in the carotenoid metabolism and flavonoid biosynthesis pathway. The expression of different transcription factors (TFs) may also influence the chlorophyll biosynthesis pathway, carotenoid metabolism and the flavonoid biosynthesis pathway in apple leaf mutants. This study provides a new method for understanding the differences in the formation of apple seedlings with different degrees of albinism.

**Supplementary Information:**

The online version contains supplementary material available at 10.1186/s12864-022-08535-3.

## Background

Photosynthesis is crucial for plant growth, development and survival [1]. Plants are a major organ of photosynthesis, and colour mutations in leaves can influence plant growth and development and even cause death, resulting in economic losses; therefore, most leaf colour mutants are removed individually. However, leaf colour mutation, also known as chlorophyll (Chl)-deficient mutation (CDM), is a common phenomenon in nature. CDMs are ideal materials for researching plant chlorophyll metabolism, photosynthesis systems, chloroplast development and genetic breeding [2, 3]*.*

Mutations in leaf colouration are always expressed at the seedling stage, and the types of CDMs are generally divided into albino, white emerald, greenish-white, yellow-green, light green, etiolation, greenish-yellow and striped [4]. These types of leaf mutants have been found in *Arabidopsis thaliana* [5–7], *Oryza sativa* [8, 9], *Zea mays* [10, 11], *Nicotiana tabacum* [12, 13], *Glycine max* [14], ginkgo [15], tea [3] and birch [16].

There are many factors that lead to CDMs. Most leaf mutants are caused by genetic changes, and greater than 700 sites are involved in mutations in leaf colouration in higher plants [17]. However, the underlying mechanism is complicated. The genetic changes in CDMs can directly or indirectly influence the synthesis, degradation, content and proportions of pigments, decreasing photosynthesis and resulting in abnormal leaf colours.

Cytosine methylation is extensively found in eukaryotes and plays a vital function in many processes, including genome integrity maintenance, transcription regulation, transposable element silencing and imprinting [18]. The methylation of DNA occurs in three different sequence contexts in plants: CG, CHG and CHH (wherein H stands for A, T or C) [19]. Many mechanisms are involved in establishing, maintaining and removing DNA methylation marks. Methyltransferase 1 (MET1) and chromomethylase 3 (CMT3) maintain methylation in the CHG and CG sequence contexts, respectively [19], whereas domain-rearranged methyltransferase 2 (DRM2) is responsible for maintaining CHH site methylation [20]. Currently, some studies involving whole-genome bisulfite sequencing (WGBS) have been reported in mutations of plant colouration associated with DNA methylation, such as apple fruit somatic mutants [21] and *Prunus mume* flower colour chimaera mutants [22], and a few reports have involved CDMs, especially apple leaf mutants.

RNA sequencing (RNA-seq) is an important technology used in the molecular research of plants. In this method, high-throughput sequencing technology is used to sequence cDNA libraries, and RNA-seq involves the reverse transcription of total RNA in tissues or cells to reflect gene expression levels. RNA-seq is also mainly used to identify plant colour mutation-related genes, and this method can determine the efficiencies and accuracies of these genes. The identification, cloning, and functional research of mutant genes and their associations with bisulfite sequencing (BS-seq) are of great significance in explaining the molecular mechanism of CDMs in plants [17].

Apples (*Malus domestica*) are extensively cultivated and economically significant perennial fruit crops. Most colour mutants focus on the colours of apple fruits [21], and few studies have examined the CDMs of apples. We used the methylome and transcriptome to analyse three different CDMs in apple rootstock G.935. Methylation and transcription analyses of apple seedlings with different degrees of albinism indicated that nine genes associated with carotenoid metabolism and the flavonoid biosynthesis pathway were differentially methylated and may be regulated by methylation changes and transcription factors (TFs). The results provide a new perspective for understanding the three different degrees of albinism in apple plants.

## Results

### Phenotypic characteristics of apple leaves

**Fig. 1:**
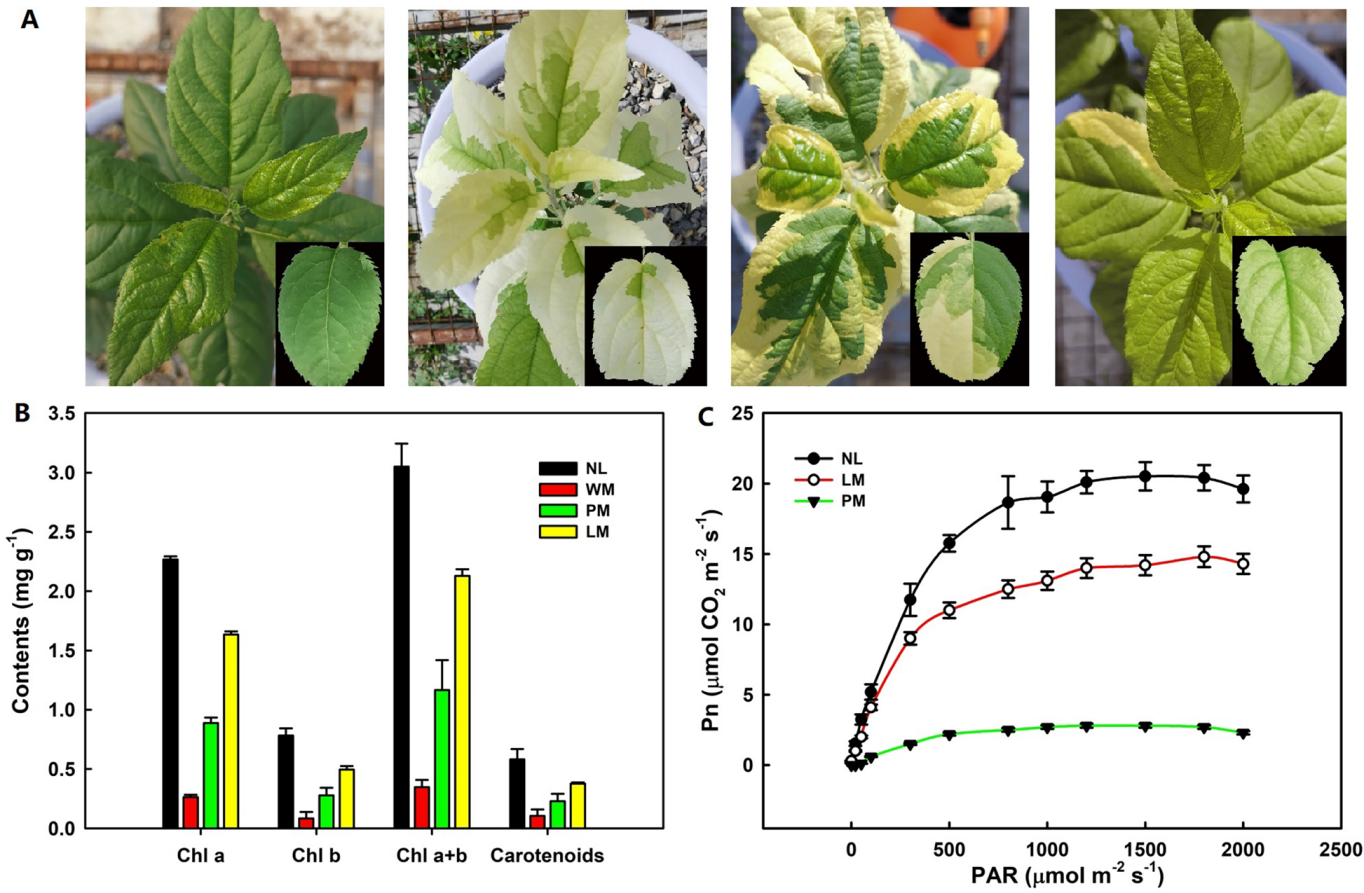
Phenotype and pigment content in normal leaves (NL), white-leaf mutants (WM), piebald leaf mutants (PM) and light-green leaf mutants (LM) of apple rootstock CG-935. **A** Phenotypes of three apple chlorophyll-deficient mutant leaves and normal leaves. **B** Chlorophyll (Chl) a, Chl b, Chl a + b and carotenoid content. **C** Responses of the net photosynthetic rate (Pn) to photosynthetically active radiation (PAR) in different apple leaves. The data are presented as the means of 3 replicates with the SD

Different CDMs of apple leaves presented significantly white, piebald and light-green leaf colours (Fig. 1A). The contents of Chl a, Chl b, Chl a + b and carotenoid in the apple leaf mutants were lower than those in normal leaves (NL) (Fig. 1B). The order of these contents was NL > light-green leaf mutants (LM) > piebald leaf mutants (PM) > white-leaf mutants (WM), demonstrating that the decreased Chl a, Chl b and carotenoid concentrations might be the key reason for the apple CDMs. As the photosynthetically active radiation (PAR) increased (Fig. 1C), the light-saturated net photosynthetic rates (Pn) of mutant leaves and normal leaves were significantly different. NL showed the highest light-saturated Pn value, and that of PM was the lowest followed by LM. The WM leaves had no measured Pn value.

### Whole-genome bisulfite sequencing of different apple leaves

According to different CDMs in the apple seedlings, we prepared three pairs of libraries (WM/NL, PM/NL and LM/NL) from different apple leaves showing different leaf mutations. In the BS-Seq analyses, approximately 10,000,000 clean reads were filtered from each sample. A total of 100,699,326, 97,804,686, 102,348,440, and 104,361,960 mapped reads were obtained (97.55%, 98.44%, 98.84% and 98.88% of the totals, respectively). The bisulfite conversion rates of the NL, WM, PM and LM leaves were 99.29%, 99.28%, 99.19% and 99.25%, respectively (Table S1). Figure S1A shows the cumulative distribution of the C-base effective sequencing depth. The fraction containing the CG, CHG and CHH sequence contexts approached 100% with the minimum depth. The fraction peaked at approximately 10 to 20 times the read depth of the additional peak at the minimum read depth (Fig. S1A), indicating that the quality of the sequencing data was high. The sequence depth distributions of the four apple leaves are described in Fig. S1B. The data in Fig. S1C indicate the relative proportions of methylcytosines (mCs) in CG, CHG and CHH sequence contexts of four apple leaves, and the mC frequencies were roughly equal; the CG, CHG and CHH sites were each approximately 30%. Based on a genome-wide perspective, the methylation landscapes of four apple genomes were apparent and are shown in Fig. S2. The mC distribution in all 17 apple chromosomes in all sequence contexts is presented in Fig. S3.

### DNA methylation patterns among different apple seedlings

**Fig. 2:**
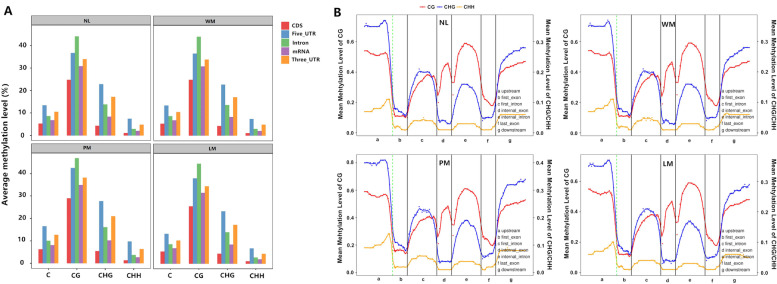
The elements of the average methylation levels and the units of whole transcription. **A** The different coverage of the elements types. Red rectangles stand for CDS, blue rectangles stand for 5’-UTR, green rectangles stand for intron, purple rectangles stand for mRNA, and orange rectangles stand for 3’-UTR. **B** The whole-genome level of DNA methylation patterns across whole transcriptional units. The canonical gene structure is defined by 7 different features, which are displayed on the x-axis. The length of features was normalised and partitioned into equal numbers of bins. Each dot denotes the mean methylation level per bin and lines denote 5-bin moving averages. The green vertical lines indicate the mean locations of transcriptional start sites. WM, PM, LM, and NL stand for white-leaf mutants, piebald leaf mutants, light-green leaf mutants, and normal leaves, respectively

The methylation profiles in gene regions were analysed to investigate the DNA methylation patterns in the mutant and wild-type apple leaves, as shown in Table S2. In each gene region of the four different leaves, the methylation level at CG was highest, followed by CHG and C (Fig. 2A). To explore the DNA methylation patterns in different apple mutant leaf genomic regions, we analysed the methylation profiles within genes. Methylation at CG was present at the highest levels in upstream regions, followed by downstream regions, introns and exons. Less methylation occurred at CG and CHH sites than at CHG sites in the upstream and downstream regions and first exons. CG sites had higher methylation levels in internal exons, introns and the last exons than CHG and CHH sites. The CHH sites showed the lowest methylation levels in all regions (Fig. 2B).

### Differentially methylated regions in different apple leaves

Differentially methylated regions (DMRs) were identified to research the extent of differential methylation among the four apple leaves. Table S3 lists the distribution of the lengths of DMRs and the number of each chromosome. A total of 61,755 DMRs were detected between WM and NL (WM/NL), 79,824 were detected between PM and NL (PM/NL), and 74,899 were detected between LM and NL (PM/NL), as shown in Table S3.

**Fig. 3:**
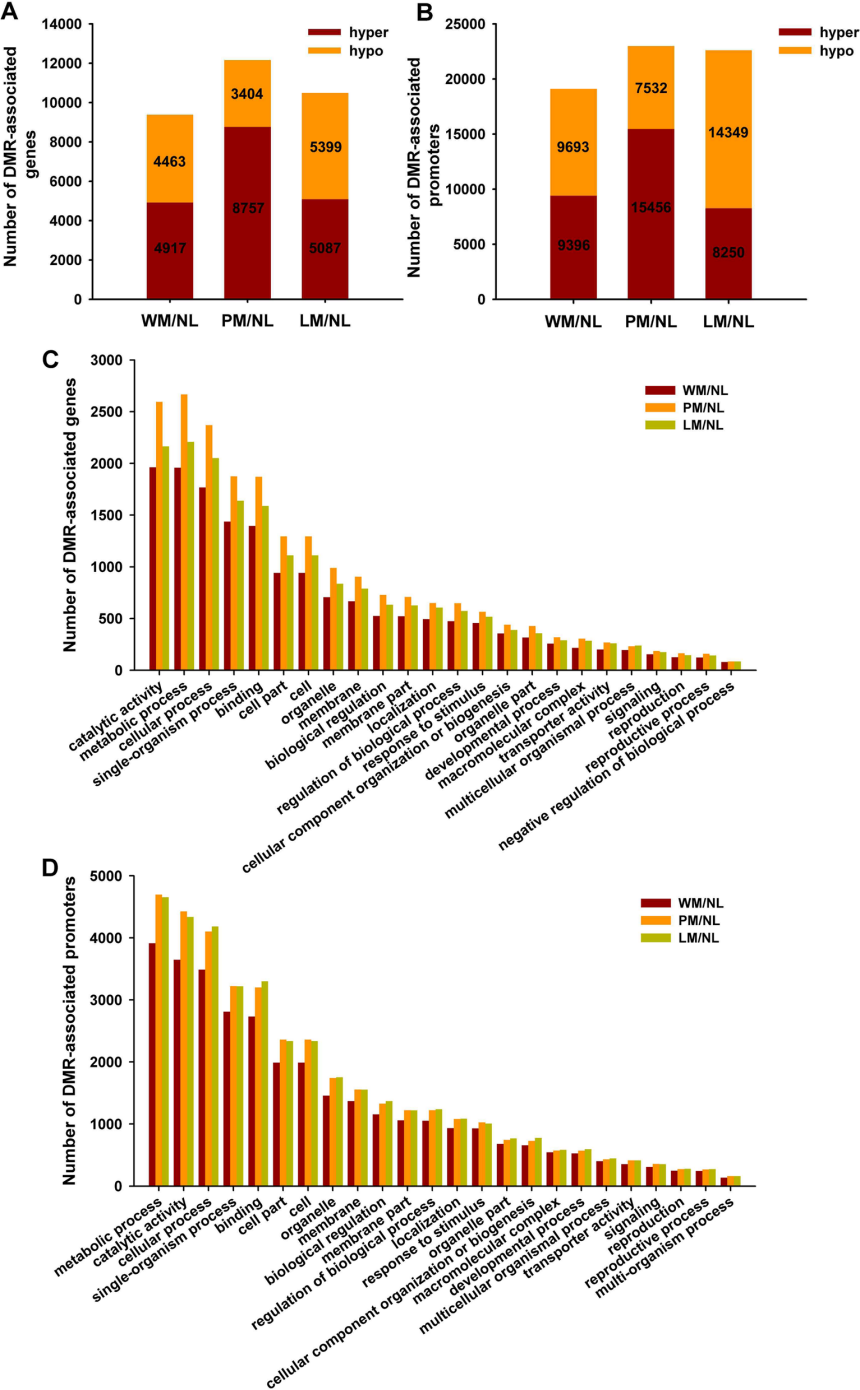
Methylation analysis of WM/NL, PM/NL and LM/NL. The number of differentially methylated region (DMR)-associated genes (**A**) and promoters (**B**) in different apple leaves. The Gene Ontology (GO) enrichment in DMR-associated genes (**C**) and promoters (**D**) in WM/NL, PM/NL and LM/NL

The DMRs were divided into DMR-associated genes and promoters, and the DMRs overlapped with the genes and promoters. A total of 9380 DMR-associated genes were found in WM/NL, 12,161 were found in PM/NL, and 10,486 were found in LM/NL (Fig. 3A). A total of 19,089 DMR-associated promoters were identified in WM/NL, 22,988 were identified in PM/NL, and 22,599 were identified in LM/NL (Fig. 3B). There were more hyper-DMR-related genes than hypo-DMR-related genes in both WM/NL and PM/NL, and PM/NL also had more hyper-DMR-related promoters than hypo-DMR-related promoters; the opposite patterns were observed in DMR-related genes and promoters of LM/NL and DMR-related promoters of WM/NL (Fig. 3B). Based on Gene Ontology (GO) analysis, DMR-associated genes were involved in different biological processes, such as catalytic activities, metabolic processes, cellular processes, single-organism processes and binding (Fig. 3C). The DMR-associated promoters revealed genes involved in metabolic processes, including catalytic activities, cellular processes, single-organism processes, binding and cell partitioning (Fig. 3D). As reflected by the analyses, the DMRs in the Kyoto Encyclopedia of Genes and Genomes (KEGG) were abundant in pathways involving flavonoid biosynthesis, flavone and flavonol biosynthesis and photosynthesis-antenna proteins (Fig. S4).

### Differential gene expression among the four apple leaves

To discover the global gene expression among the different apple leaves, RNA-seq analyses were performed for the four apple leaves (each leaf was assessed using 3 biological replicates). A summary of the sequencing data is listed in Table S4. Principal component analysis (PCA) showed that the NL, LM, PM and WM samples were separately aggregated, indicating that significant differences existed in the gene expression profiles. The four leaves replicates did not strictly cluster together, suggesting that inoculation within the replicates differed (Fig. S5). After removing low-quality reads, the differentially expressed genes (DEGs) were identified with a fold change ≥1 and *p*-value < 0.05. Fig. S6A, B, C shows the volcano plot of DEGs in WM/NL, PM/NL and LM/NL. A total of 7566 (2462 upregulated and 5104 downregulated), 3660 (869 upregulated and 2791 downregulated), and 3546 (1906 upregulated and 1640 downregulated) transcripts were differentially expressed in the WM/NL, PM/NL and LM/NL comparisons, respectively (Fig. S6D).

In the GO analysis, the DEGs were divided into 37 functional terms, such as biological process, cellular component and molecular function. Most GO term genes of those functional terms were downregulated in WM/NL and PM/NL, and upregulated in LM/NL. The biological process genes were enriched in metabolic, cellular and single-organism processes (Fig. S7A). Regarding their biological functions, KEGG analysis showed that the DEGs were observably involved in flavonoid biosynthesis, DNA replication, metabolic pathways and secondary metabolite biosynthesis pathways (Fig. S7B).

**Fig. 4:**
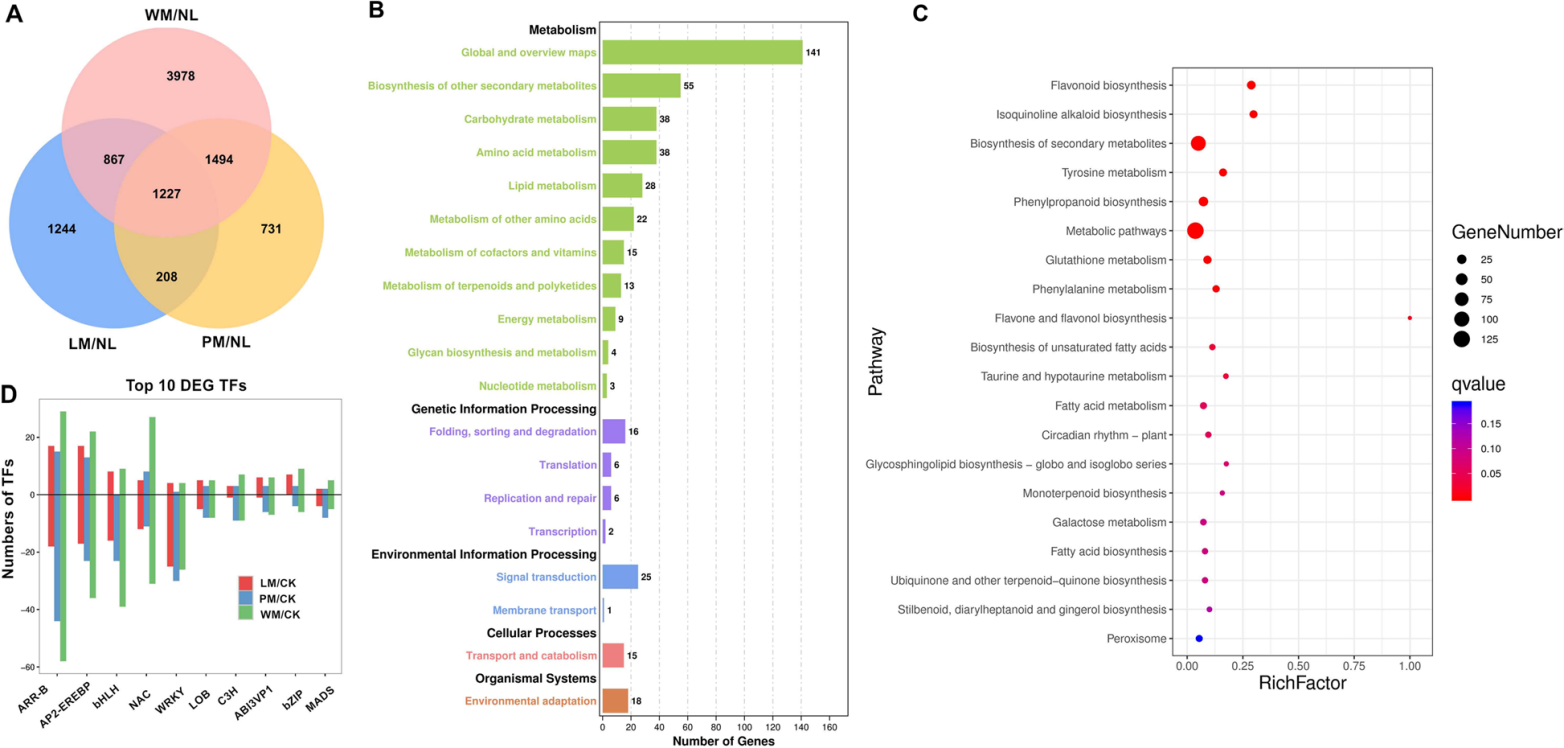
Differential gene expression (DEGs) and Gene Ontology (GO) analysis in WM/NL, PM/NL and LM/NL. **A** The number of DEGs found in the comparisons of WM/NL, PM/NL and LM/NL are presented as a Venn diagram. **B** GO enrichment analysis of DEGs in WM/NL, PM/NL and LM/NL. **C** KEGG pathway analysis of DEGs in the WM/NL, PM/NL and LM/NL comparisons. **D** Top 10 number of DEGs associated with transcription factors (TFs) in WM/NL, PM/NL and LM/NL. Positive and negative values of the y-axis indicate the number of up- and downregulated transcription factors

Among the DEGs, a total of 1227 DEGs were found in all comparisons (Fig. 4A), which were involved in metabolic processes, genetic information processing, environmental information processing, cellular processes and organismal systems (Fig. 4B). Enrichment analysis of the DEGs showed that these genes were significantly enriched in metabolic pathways, secondary metabolite biosynthesis and flavonoid biosynthesis (Fig. 4C). Additionally, 517 DEGs of TFs were found in WM/NL, 290 DEGs of TFs in PM/NL, and 267 DEGs of TFs in LM/NL. The most highly represented TFs included the *ARR-B*, *AP2-EREBP*, *bHLH*, *NAC*, *WRKY*, *LOB*, *C3H* and *ABI3VP1* families (Fig. 4D). For example, *MdMYB4* (MD16G1218000) expression was decreased 3.55-, 8.29- and 2.31-fold, and *MdbHLH47* (MD06G1191600) expression was decreased 3.13-, 2.99- and 2.88-fold in WM/NL, PM/NL and LM/NL, respectively.

### Analysis of the DEGs related to the chlorophyll biosynthesis pathway, carotenoid metabolism and flavonoid biosynthesis pathway

**Fig. 5:**
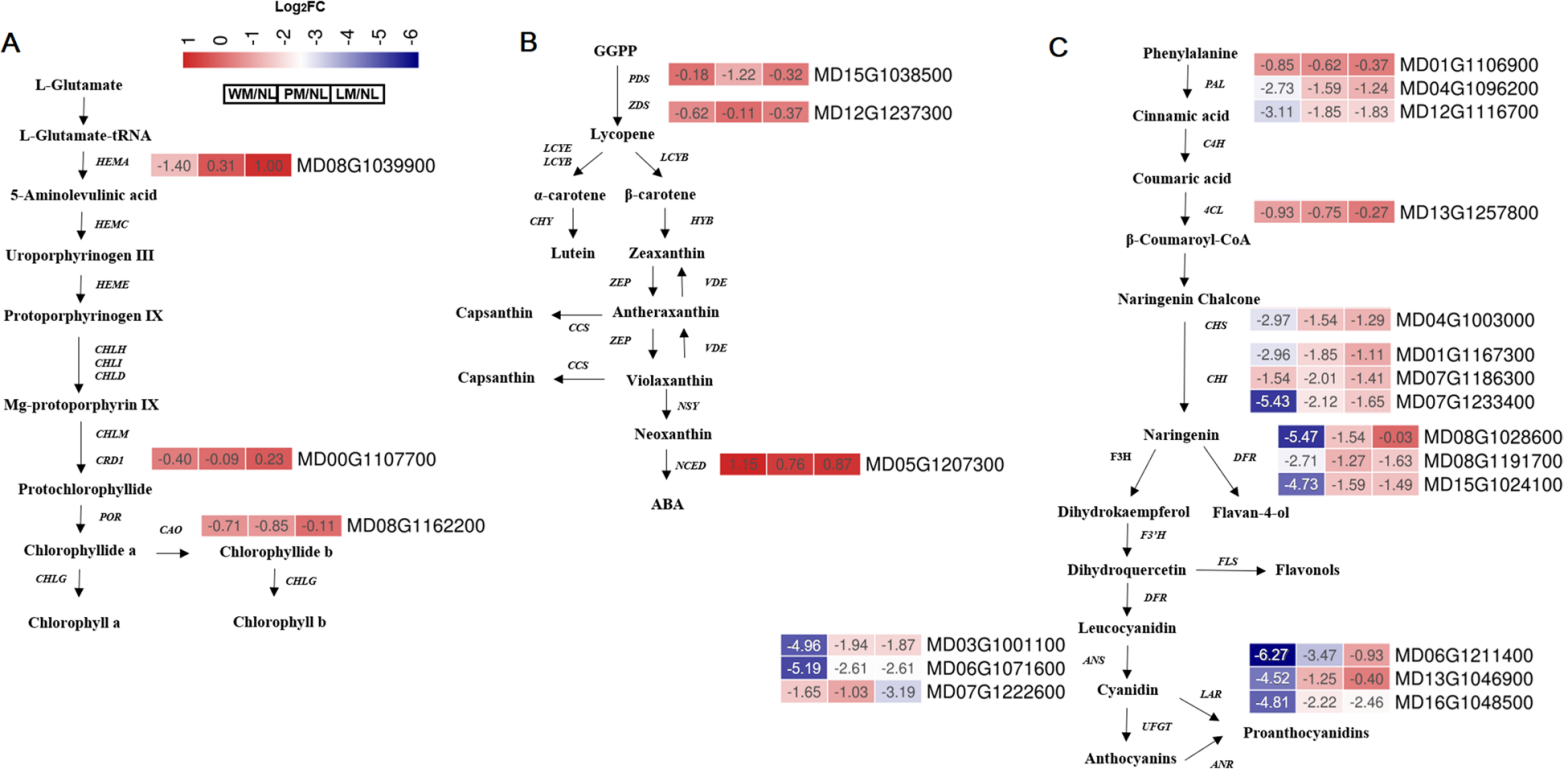
DEGs related to the three pigment biosynthesis pathways. **A** The chlorophyll biosynthesis pathway. **B** The carotenoid biosynthesis pathway. **C** The flavonoid biosynthesis pathway. The green and red rectangles represent the decreased and increased levels of DEGs, respectively. The boxes represent WM/NL, PM/NL and LM/NL

Leaf pigments, such as chlorophyll, carotenoids and flavonoids, can influence plant leaf colouration [3]. Therefore, the DEGs involved in these pathways were selected to investigate differential gene expression among different leaf colourations (Table S5), including the following: *HEMA* (MD08G1039900), *CRD1* (MD00G1107700) and *CAO* (MD08G1162200) genes involved in chlorophyll biosynthesis pathways (Fig. 5A); *PDS* (MD15G1038500), *ZDS* (MD12G1237300) and *NCED* (MD05G1207300) in carotenoid biosynthesis pathways (Fig. 5B); and *PAL* (MD01G1106900, MD04G1096200 and MD12G116700), *4CL* (MD13G1257800), *CHS* (MD04G1003000), *CHI* (MD01G1167300, MD07G1186300 and MD07G1233400), *DFR* (MD08G1028600, MD08G1191700 and MD15G1024100), *LAR* (MD06G1211400, MD13G1046900 and MD16G1048500) and *ANS* (MD03G1001100, MD06G1071600 and MD07G1222600) in flavonoid biosynthesis pathways (Fig. 5C).

The green leaf colouration is due to chlorophyll accumulation. Chlorophyllide and oxygenase (*CAO*) gene expression was decreased 1.42-, 1.69- and 0.21-fold in WM, PM and LM, respectively, compared with that in NL. The synthesis of 5-aminolevulinic acid (ALA) is a rate-limiting step in chlorophyll biosynthesis. Reduced expression of the synthetic gene glutamyl-tRNA reductase (*HEMA*) may affect chlorophyll metabolism in WM and LM. Mg-protoporphyrin IX monomethyl ester (*CRD1*) gene expression was upregulated in the mutant apple leaves in WM and PM. The transcription factor *MdMYB308* (MD07G1153200) was downregulated in three mutant apple leaves. Therefore, the downregulated genes might influence the chlorophyll metabolism of mutant apple leaves.

In carotenoid metabolism, the phytoene desaturase (*PDS*) (MD15G1038500) and ζ-carotene desaturase (*ZDS*) (MD12G1237300) genes were more highly expressed in WM, PM and LM compared with NL. Interestingly, the rate-limiting enzyme 9-cis-epoxycarotenoid dioxygenase (NCED) regulates carotenoid conversion to abscisic acid (ABA). *NCED* (MD05G1207300) gene expression was upregulated 4.60-, 3.00- and 3.48-fold in WM/NL, PM/NL and LM/NL, respectively (Fig. 5B), which might cause the degradation of carotenoids.

In the flavonoid synthesis pathway, the expression of phenylalanine ammonia-lyase (*PAL*) (MD04G1096200) was decreased 5.46-, 3.66- and 2.48-fold compared with NL in the three mutants, and *PAL* (MD12G1116700) was decreased 6.22-, 2.70- and 2.66-fold compared with NL (Fig. 5C). Chalcone synthase (*CHS*) (MD04G1003000), chalcone isomerase (*CHI*) (MD01G1167300, MD07G1186300 and MD07G1233400), dihydroflavonol-4-reductase (*DFR*) (MD08G1028600, MD08G1191700 and MD15G1024100), leucoanthocyanidin reductase (*LAR*) (MD06G1211400, MD13G1046900 and MD16G1048500) and leucoanthocyanidin dioxygenase (*ANS*) (MD03G1001100, MD06G1071600 and MD07G122600) were also decreased in the three mutant apple leaves. The transcription factor *MdbHLH3* (MD11G286900) combines with the *DFR* gene to regulate the flavonoid synthesis pathway [23], and *MdbHLH3* expression levels in WM/NL and PM/NL were decreased 2.82- and 2.13-fold, respectively. The TT2 transcription factor (MD09G1184000) is an R2R3 MYB domain protein that regulates *LAR* [24], and its expression was decreased 1.74-, 1.63- and 1.75-fold in WM/NL, PM/NL and LM/NL, respectively. These results potentially explain the decreased *DFR* and *LAR* gene expression in the mutants. Some MYBs control anthocyanin production in plants via the transcriptional regulation of structural genes (Naing and Kim, 2018), and the transcription factors *MYB308* (MD06G1229700, MD07G1153200, MD14G1234500 and MD14G1234600) and *MYB4* (MD16G1218000) were also decreased in three CDMs apple leaves.

### Correlation analysis between DMRs and DEGs in different apple leaves

**Fig. 6:**
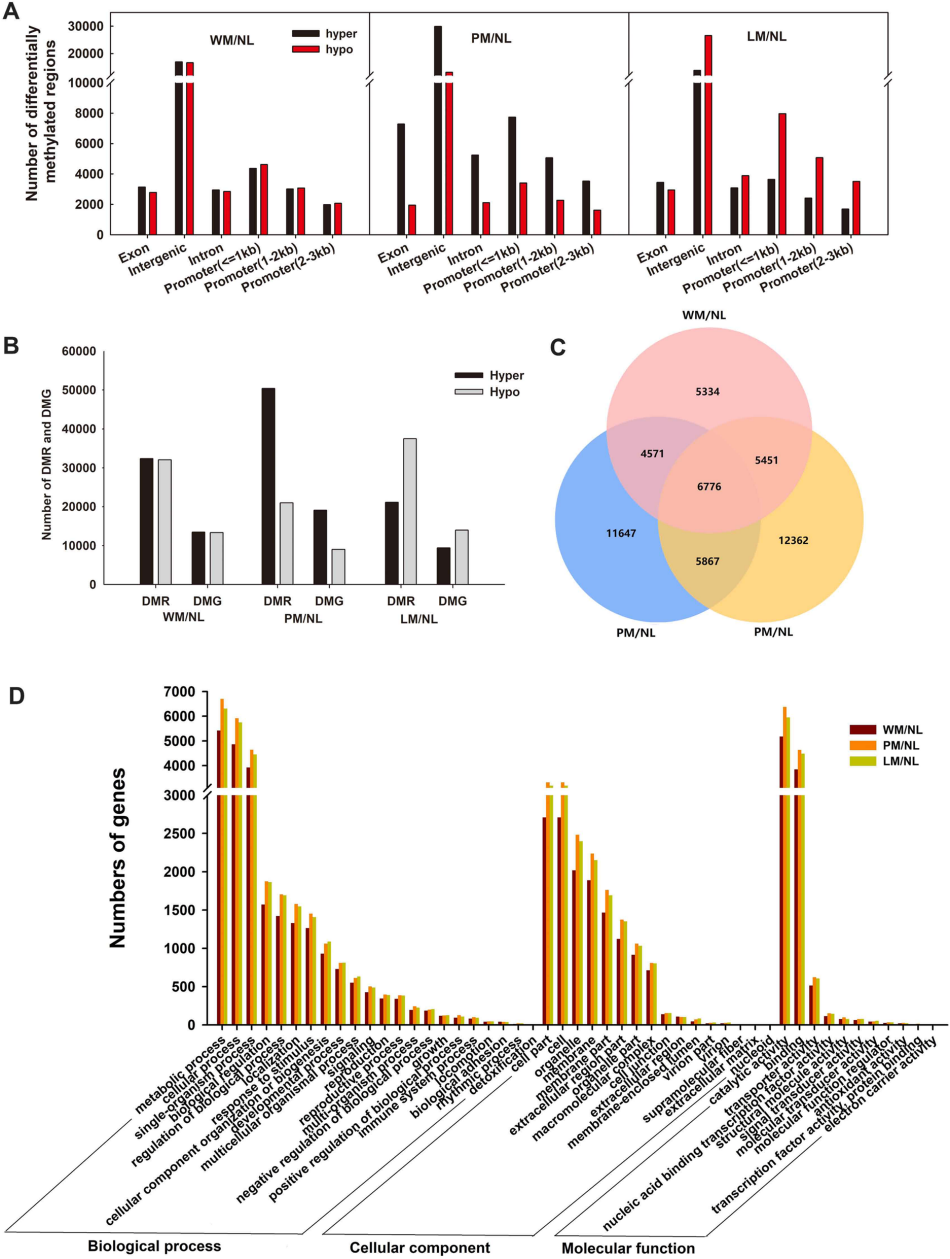
Whole-genome methylome analysis of WM/NL, PM/NL and LM/NL. **A** Numbers of differentially methylated regions (DMRs) among WM/NL, PM/NL and LM/NL. DMRs involved in intergenic and genic regions. The genic regions include exons, introns, and promoters. **B** DMRs and differentially methylated genes (DMGs) in WM/NL, PM/NL and LM/NL. **C** Venn diagrams of DMRs in WM/NL, PM/NL and LM/NL. **D** Gene Ontology (GO) analysis of DMGs in WM/NL, PM/NL and LM/NL

The correlation between DMRs and DEGs on the whole-genome scale was assessed to investigate the influence of DNA methylation on gene expression. A total of 263,066 DMRs were found between the mutant leaves and the normal leaves. Approximately 51.89% of the DMRs were distributed in the distal intergenic regions, and 30.38% of the DMRs were found in promoter regions (Fig. 6A). The number of hyper-DMRs was approximately equal to the number of hypo-DMRs in WM/NL, and the number of hyper-DMRs was higher than that of hypo-DMRs in PM/NL and the opposite in LM/NL (Fig. 6A). In WM/NL, 74,840 DMRs (37,717 hypermethylated and 37,123 hypomethylated DMRs) were found, and these DMRs overlapped with 16,033 hypermethylated and 15,858 hypomethylated genes. In PM/NL, 98,020 DMRs (70,511 hypermethylated and 27,509 hypomethylated DMRs) were selected and overlapped with 27,552 hypermethylated and 11,973 hypomethylated genes. In LM/NL, 90,206 DMRs (31,708 hypermethylated and 58,498 hypomethylated DMRs) were found, and these regions overlapped with 14,258 hypermethylated and 22,580 hypomethylated genes (Fig. 6B).

By combining the results obtained from the different mutants, 52,008 and 6776 differentially methylated genes (DMGs) were identified (Fig. 6C). GO analysis was performed on the DMGs to understand the possible role of DNA methylation in the different apple leaves. These results indicated notable differences in biological processes, cellular components and molecular functions during the greening stage (Fig. 6D). Figure S8 shows the number of DMGs at CG, CHG, and CHH sites between DMRs and DEGs in WM/NL, PM/NL and LM/NL. Methylation at CHH sites was noted in most genes between DMRs and DEGs, followed by methylation at CG and CHG sites. Figure S9 indicates the gene expression patterns and methylation levels. Although the expression levels of hyper-DMR genes and all genes were slightly higher than the expression levels of hypo-DMR genes in the expression of DMR-associated genes and promoters, there was no statistically significant difference between hypomethylated or hypermethylated genes and all genes. There was also no obvious difference in the hypermethylated or hypomethylated with DEG-associated genes or promoters among the three different mutant comparisons.

### DMR-associated DEGs are involved in the carotenoid and flavonoid biosynthesis pathways

We also focused on the DMR-associated DEGs involved in the chlorophyll, carotenoid and flavonoid biosynthesis pathways (Fig. 5 and Table S6). There were no DMR-associated DEGs involved in the chlorophyll biosynthesis pathways. In the carotenoid biosynthesis pathway, *PDS* (MD15G1038500) gene expression was decreased in mutant leaves compared with normal leaves, and the *PDS* promoter was hypermethylated in PM/NL. In the flavonoid biosynthesis pathway, the *PAL* (MD04G1096200) gene exhibited decreased expression and a hypermethylated gene body in PM/NL. In WM/NL, LM/NL and PM/NL, *CHS* (MD04G1003000) was downregulated with a hypermethylated promoter. In WM/NL, LM/NL and PM/NL, *CHI* (MD07G1186300 and MD07G1233400) exhibited decreased expression and a hypermethylated gene body. In PM/NL, *CHI* (MD01G1167300) exhibited decreased expression and hypermethylated gene bodies. The expression of *DFR* (MD15G1024100) was also decreased, and its gene body and promoter were hypermethylated in PM/NL. In PM/NL and LM/NL, *LAR* (MD16G1048500) expression was downregulated with a hypermethylated gene body. In PM/NL, *ANS* (MD03G1001100) downregulated gene with a hypermethylated promoter was observed.

### Confirmation of DNA methylation and gene expression data

Several DMR genes (MD07G12334000, MD15G1024100, MD04G1003000, MD03G1001100 and MD13G1257800) involved in the carotenoid and flavonoid biosynthesis pathways were selected for bisulfite-PCR and sequencing analyses. Using four apple leave samples, these regions were subjected to bisulfite-PCR and then sequenced. The bisulfite-PCR results were consistent with the corresponding methylome analysis (Fig. S10), indicating that the sequencing results were reliable.

To validate the RNA-seq results, 15 DEGs (MD04G1096200, MD12G1116700, MD04G1003000, MD01G1167300, MD07G1186300, MD07G1233400, MD08G1191700, MD15G1024100, MD03G1001100, MD08G1207100, MD16G1003400, MD14G1147900, MD05G1207300, MD13G1004100 and MD16G1048500) involved in the chlorophyll, carotenoid and flavonoid biosynthesis pathways were validated at the transcript level in four apple leaves. The RT-qPCR results were not significantly different from the RNA-seq data, and similar trends were found in the up- and downregulated genes (Fig. S11).

## Discussion

Colour mutation in leaves is an easily identifiable and common phenomenon in higher plants. CDMs frequently affect photosynthetic plant efficiency, causing plant growth delay and economic losses. For this reason, CDMs have been negatively selected in the past. More recently, the use of CDMs has become more common. Mutants are ideal materials for researching chloroplast differentiation and development, pigment metabolism, photosynthesis and other pathways; mutants also supply significant information for improving varietal selection [17].

Apples are a widely cultivated and economically significant perennial fruit. Most research has focused on the colour of apple flesh [21], and the CDMs of apples are rarely involved. In this study, we analysed differences in the methylome analyses and transcriptome analyses of three different natural CDMs of G.935 apple. We provide a reference for future research and application of CDM.

In plants, the DNA methylation machinery may affect growth, development, the regulation of biosynthesis and phenotypic plasticity [25–27]. Therefore, it is important to explain the differences in the methylomes of DNA between CDMs and normal apple leaves to understand epigenetic regulation. The DNA methylation dynamics were investigated at a single-base resolution through WGBS in four apple leaves (NL, WM, PM and LM). The genome showed methylation levels of 62.85–67.00%, 44.52–49.25%, and 6.73–9.05% at the CG, CHG and CHH sites, respectively (Table S7). The methylation of CG was found to be more common than the methylation of CHG or CHH sites in previous methylome studies of apple plants [21]. In the four apple leaves studied here, methylation at CG, CHG and CHH sites followed the same trend; methylation was the most common at CG sites, followed by CHG and CHH sites. Among the four leaves, PM had the highest methylation levels, and NL had the lowest methylation levels. This finding indicated that the methylation level in the normal leaves was relatively stable; on the other hand, the PM leaves exhibited substantial changes in their methylation levels. Previous research indicated that the level of DNA methylation positively correlates with genome size [28, 29]. Compared with other plants, such as *Arabidopsis*, *Eutrema salsugineum*, *Vitis vinifera* and *Beta vulgaris*, apple plants show middling methylation levels [29]. These results indicated that DNA methylation primarily functions to maintain genome stability [21].

As an epigenetic marker, DNA methylation plays a role in repressing gene expression. When first conducting genome-wide methylome analyses, the relationship between DNA methylation and gene expression was found to be more complicated than initially believed [3]. For example, in rice and *Arabidopsis*, promoter methylation restrains the expression of genes only at highly methylated gene loci, and gene body methylation is always positively related to gene expression [30, 31]. Global methylation and transcriptional analyses indicated that nonexpressed genes or genes expressed at low levels were highly methylated in the gene body regions, suggesting that gene body methylation negatively correlates with gene expression. Moreover, genes with methylated promoters were more highly expressed than genes with unmethylated promoters, implying that promoter methylation and gene expression are positively correlated [32].

For certain DMR-associated DEGs, the opposite pattern was observed. In the carotenoid biosynthesis pathway, the *PDS* gene (MD15G1038500) was downregulated with hypomethylated promoters in PM/NL. The analyses also emphasized eight DMR-associated downregulated DEGs related to the flavonoid biosynthesis pathway. Specifically, *PAL* (MD04G1096200) exhibited decreased expression and a hypomethylated gene body in PM/NL. In WM/NL, LM/NL and PM/NL, *CHS* (MD04G1003000) represented downregulated genes with hypermethylated promoters. In WM/NL, LM/NL and PM/NL, *CHI* (MD07G1186300, MD07G1233400) exhibited decreased expression and a hypomethylated gene body. In PM/NL, *CHI* (MD01G1167300) was decreased and had a hypomethylated gene body. *DFR* (MD15G1024100) also showed decreased expression with a hypermethylated gene body and promoter in PM/NL. In PM/NL and LM/NL, *LAR* (MD16G1048500) exhibited downregulated expression with a hypermethylated gene body, and *ANS* (MD03G1001100) exhibited downregulated expression with a hypomethylated promoter. In general, the expression of many genes was not associated with the corresponding methylation level. Although some down- and upregulated genes were related to hyper- and hypo-DMRs, many DEGs did not show remarkably different methylation levels. DNA methylation influences the expression of genes and is likely influenced by a combination of direct and indirect mechanisms [21]. The bisulfite-PCR and RT-qPCR results were consistent with the corresponding methylome analysis and RNA-seq data (Fig. S10 and S11), indicating that the sequencing results were reliable.

Plant leaf colouration is also influenced by the pigments in leaves, such as chlorophyll, carotenoids and flavonoids [3]. Leaf colouration is also affected by the chlorophyll, carotenoid and flavonoid biosynthesis pathways. In plant leaves, the chlorophyll and carotenoid contents are the main colouring matter that capture light energy. The colouration of plant leaves is mainly influenced by the biosynthesis and transport of chlorophyll and carotenoids [15, 33]. Many albino leaves have conspicuously decreased chlorophyll levels [34, 35]. In this research, less Chl a and Chl b were detected in the CDMs than in NL (Fig. 1A). Chl b biosynthesis regulates light-harvesting efficiency and photosynthetic antenna size [36]. The decreased chlorophyll contents observed in the CDMs indicate that the mutants might have less light-harvesting efficiency than NL and that this factor may affect leaf colouration. The expression of *CAO, CRD1, HEMA* and the transcription factor *MdMYB308* was decreased, which may induce low Chl b levels in the three mutant leaves (Fig. 5A). *MdMYB308* (MD07G1153200) was also downregulated in three mutant apple leaves. The homologous gene *CsMYB36* (Csa2G352940) is involved in the formation of the yellow-green peel in *Cucumis sativus* [37], and a *CsMYB36* mutation could affect the synthesis of L-glutamate-tRNA to 5-aminopentanone, resulting in low chlorophyll levels in the pericarps of yellow-green peel mutants and affecting the chlorophyll content.

The pigments of carotenoids are red, orange, or yellow. Carotenoid biosynthesis is balanced with chlorophyll synthesis in the chloroplast, and carotenoids are also essential for photoprotection [38]. Various carotenoids exhibit changes in composition, especially displaying markedly increased zeaxanthin levels, leading to albino tea cultivars [35] and yellow-leaf mutants of winter wheat [33]. The total carotenoid contents in NL, WM, PM and LM were obviously different, and the carotenoid contents in mutants were significantly decreased compared with those in normal leaves (Fig. 1A). In particular, NCED is a rate-limiting enzyme that controls carotenoid transformation to ABA, and *NCED* gene expression was upregulated in WM, PM and LM (Fig. 5B) and may influence carotenoid degradation and contribute to carotenoid component changes.

Flavonoids, including anthocyanins, flavones and flavonols, also affect leaf colouration [15]. Structural gene expression in the flavonoid metabolic pathway can influence plant colouration. Under light induction, the activity of PAL and anthocyanin levels increase in purple-foliage plum leaves, and the colour of leaves gradually turns purplish red [39]. CHS is also a key enzyme in the anthocyanin biosynthetic pathway. When the *CHS1* gene of *Freesia hybrida* was incorporated into *Petunia hybrida* as a transgene, the colour of *P. hybrida* changed from white to pink [40]. Silencing of the *CHI* gene can induce yellow pigmentation in tobacco and carnation [41]. Moreover, the oxidation of downstream colourless proanthocyanin is regulated by ANS into the coloured anthocyanin. The genes and transcription factors in the flavonoid metabolic pathway, including *PAL*, *4CL*, *CHS*, *CHI*, *DFR*, *LAR, ANS* and *MdbHLH3* were decreased in apple with different CDMs (Fig. 5C). The increased methylation of these genes and the regulation of transcription factors may be responsible for the downregulation of the genes and influence the phenotypes of apple leaves.

## Conclusions

The methylomes and transcriptomes of apple seedlings with three different degrees of albinism were analysed, and DMR-associated DEGs in carotenoid metabolism and the flavonoid biosynthesis pathway were identified in three comparisons (WM/NL, PM/NL and LM/NL). The DEGs included *PDS* (MD15G1038500), *PAL* (MD04G1096200), *CHS* (MD04G1003000), *CHI* (MD01G1167300, MD07G1186300, MD07G1233400), *DFR* (MD15G1024100), *LAR* (MD16G1048500) and *ANS* (MD03G1001100), and had hypermethylated promoter or gene body regions. The expression of different transcription factors may also influence the chlorophyll, carotenoid and flavonoid biosynthesis pathways. The differential methylation of structural gene promoters and transcription factors affected different biosynthesis pathways in the apple leaf mutants, which may explain the induction of white-leaf mutants, piebald leaves and light-green leaves in apple leaves.

## Methods

### Plant materials and growth conditions

Seedlings of apple rootstock G.935 with different degrees of albinism selected by natural variability and wild type G.935 were used in this study. G.935 plants with three different degrees of mutation and wild-type plants were grown at 25 °C in a greenhouse with a 16-h light/8-h dark photoperiod for two months. The virescent leaves of the mutant plant were named light-green leaves (LM), the zebra leaves were named piebald leaf mutants (PM), and the albino leaves were named white-leaf mutants (WM). Wild-type G.935 was treated as a control and termed normal leaves (NL). After the determination of net photosynthesis, the chlorophyll contents and carotenoid contents, the mature leaves from the three mutants and the NL at 9 to 12 positions along the stem base were sampled between 10 and 11 am, immediately frozen in liquid nitrogen, and used for the WGBS and RNA-seq analysis.

### Photosynthetic measurements

Leaves were collected from 4 different apple plants to detect different physiological indexes. Ninety-five percent ethyl alcohol was used to extract Chl a, Chl b, Chl a + b and carotenoids, and the concentrations were determined spectrophotometrically as described by Arnon [42].

The Pn of apple leaves was monitored on sunny days as described by Sun et al. [43]. PAR was gradually decreased stepwise using an integrated light-emitting diode (LED) light source from 2000 to 0 μmol m^− 2^ s^− 1^, and the Pn was recorded at each PAR point when it was stable.

### WGBS analysis

Apple DNA genomes were extracted from mutant and wild-type leaves using a Plant Genomic DNA Purification Kit (Tiangen, Beijing, China). Sonication was performed to fragment the DNA into 100- to 300-bp fragments (Covaris, MA, USA). The genomic fragments were ligated with adapters, converted with bisulfite using a Methylation-Gold kit (Zymo, CA, USA), and sequenced by using an Illumina HiSeq™2500 from Gene Denovo Biotechnology Co. (Guangzhou, China).

For the data analysis, BSMAP software was used to map the clean reads back to the apple reference genome. The sliding-window approach with a 200-bp window slide at 50-bp intervals was used for each sequence context to identify the DMRs. DMRs for each sequence context (CG, CHG and CHH) between sample 1 and sample 2 were determined according to different criteria: at least five methylated cytosine sites; coverage of more than ten reads; distance between adjacent methylated sites < 200 bp; methylation level of greater than 20%; and Fisher’s exact test *p*-values < 0.05 and false discovery rate (FDR) < 0.05 using Methylkit (V1.4.1) [44]. The DMR-associated genes and promoters were characterized after the DMRs were identified. Gene regions were classified into the promoter (2 kb upstream from transcription start sites), exon, intron, upstream (up to 2 kb gene start) and downstream (up to 2 kb gene end) regions.

GO analysis was performed with GO::Term Finder software [45] and the KEGG enrichment tool (http://www.kegg.jp/kegg/) [46] to study the DMR-associated genes and promoters. Bonferroni correction was used to calculate the *p*-value. GO terms with corrected *p*-values < 0.05 were regarded as greatly enriched by DMR-associated genes or promoters. KEGG pathways with *p*-values < 0.05 were considered significantly enriched with DMR-related genes.

### RNA extraction and the transcriptome sequencing

The total RNA of the apple leaves was separated using TRIzol reagent (Invitrogen, Carlsbad, CA, USA) for transcriptome analysis, and three biological replicates were used for each sample. For library construction, 5 μg of RNA per sample was used. The libraries were sequenced using an Illumina HiSeq2500 from Gene Denovo Biotechnology Co. (Guangzhou, China). After rapid filtering [47] (version 0.18.0), the clean reads were compared with the apple genome (https://iris.angers.inra.fr/gddh13/index.html) by HISAT2.2.4 and Bowtie2 tools [48, 49]. For each transcription region, the fragment per kilobase of transcript per million mapped reads (FPKM) values were calculated using RESM software [50]. DESeq2 software was used to identify the DEGs [51] based on a fold change ≥2 and divergence probability ≥0.8 [52]. The DEGs were analysed according to the default parameters by GO and KEGG enrichment, and GO terms with corrected *p*-values < 0.05 were considered significantly enriched by DEGs.

### Bisulfite sequencing PCR (BSP) analysis

Samples of gDNA (750 ng) from different apple leaves, including WM, PM, LM and NL, were treated with an EZ DNA Methylation-Gold Kit (Zymo Research) according to the method of Jiang et al. [21]. Each fragment was subjected to three independent PCRs to produce 12 independent clones for sequencing. The online software Kismeth was used to analyse the results (http://katahdin.mssm.edu/kismeth). The primers used for BSP-PCR are listed in Table S8.

### Reverse transcription and qRT-PCR

A Revert Aid First Strand cDNA Synthesis Kit (Thermo Scientific, Waltham, MA, USA) was used to reverse transcribe 1 μg RNA with a CFX96 instrument (BioRad, Hercules, CA, USA) and SYBR® Premix Ex Taq™ II (Takara, Dalian, China) to perform quantitative real-time reverse transcriptase PCR (qRT-PCR). The gene elongations factor 1α in *M. domestica* (*EF-1*α; DQ341381) was used to standardize different genes in the cDNA samples [53]. The 2^-ΔΔCT^ method was used to compute the relative expression level of each gene [54]. Three biological samples were used in all experiments. The primers used for qRT-PCR are listed in Table S8.

### Statistical analysis

Three biological and three technical replicates were included in all experiments. The data are presented as the mean ± SD. The significant differences among groups were evaluated using Duncan’s multiple-range test. The *p*-values < 0.05 were considered statistically significant.

## Supplementary Information

**Additional file 1: Table S1.** Information on the bisulfite sequencing libraries. **Table S2.** The coverage of the intergenic region. **Table S3.** The DMRs distributed on each chromosome. **Table S4.** RNA-seq sequencing data and mapping of the genome. **Table S5.** Differentially expressed genes involved in the flavonoid, chlorophyll and carotenoid biosynthesis pathways in WM/NL, PM/NL and LM/NL. **Table S6.** DMR-associated DEGs involved in the carotenoid and flavonoid biosynthesis pathways. **Table S7.** The average methylation levels in NL, WM, PM and LM. **Table S8.** The primers used in qRT-PCR and BS-PCR. **Fig. S1.** Information of the sequencing and mapping. **Fig. S2.** The global methylome in NL, WM, PM and LM. **Fig. S3.** The chromosomal mCs distributions in NL, WM, PM and LM. **Fig. S4.** The KEGG analysis of DMRs in WM/NL, PM/NL and LM/NL. **Fig. S5.** The principal component analysis (PCA) of expressed genes. **Fig. S6.** DGEs in WM/NL, PM/NL and LM/NL. **Fig. S7.** GO and KEGG pathway analyses of the DEGs in WM/NL, PM/NL and LM/NL. **Fig. S8.** Number of differentially methylated genes of CG, CHG and CHH between DMRs and DEGs in WM/NL, PM/NL and LM/NL. **Fig. S9.** Relationship between differential methylation and gene expression. **Fig. S10.** DNA methylation validated in WM/NL, PM/NL and LM/NL. **Fig. S11.** Gene expression validation involved in the chlorophyll, carotenoid and flavonoid biosynthesis pathways.

## Data Availability

The methylome data and transcriptome data have been deposited into the National Center for Biotechnology Information (NCBI) databases, and the bioproject accession number is PRJNA705949 (https://www.ncbi.nlm.nih.gov/sra/PRJNA705949).

## Abbreviations

Chl: Chlorophyll
MET1: Methyltransferase 1
CMT3: Chromomethylase 3
DRM2: Domain-rearranged methyltransferase 2
WGBS: Whole-genome bisulfite sequencing
PAR: Photosynthetically active radiation
Pn: Net photosynthetic rates
mCs: Methylcytosines
GO: Gene ontology
KEGG: Kyoto encyclopedia of genes and genomes
PCA: Principal component analysis
CAO: Chlorophyllide an oxygenase
ALA: Synthesis of 5-aminolevulinic acid
HEMA: The synthetic gene glutamyl-tRNA reductase
CRD1: Mg-protoporphyrin IX monomethyl ester
PDS: Phytoene desaturase
ZDS: ζ-carotene desaturase
NCED: Rate-limiting enzyme 9-cis-epoxycarotenoid dioxygenase
ABA: Abscisic acid
PAL: Phenylalanine ammonia-lyase
CHS: Chalcone synthase
CHI: Chalcone isomerase
DFR: Dihydroflavonol-4-reductase
LAR: Leucoanthocyanidin reductase
ANS: Leucoanthocyanidin dioxygenase

## References

[1] Li J, Chen F, Li Y, Li P, Wang Y, Mi G, et al. ZmRAP2.7, an AP2 transcription factor, is involved in maize brace roots development. Front Plant Sci. 2019;10:820. 10.3389/fpls.2019.00820 PMC6621205 31333689

[2] Larkin RM, Alonso JM, Ecker JR, Chory J (2003). GUN4, a regulator of chlorophyll synthesis and intracellular signaling. Science..

[3] Wang PJ, Zheng YC, Guo YC, Liu BS, Jin S, Liu SZ, et al. Widely targeted metabolomic and transcriptomic analyses of a novel albino tea mutant of “Rougui”. Forests. 2020;11(2):299.

[4] Wei C, Yang H, Wang S, Zhao J, Liu C, Gao L (2018). Draft genome sequence of *Camellia sinensis* var. sinensis provides insights into the evolution of the tea genome and tea quality. P Natl Acad Sci USA.

[5] Aluru MR, Yu F, Fu A, Rodermel S (2006). *Arabidopsis* variegation mutants: new insights into chloroplast biogenesis. J Exp Bot.

[6] Moon J, Zhu L, Shen H, Huq E (2008). PIF1 directly and indirectly regulates chlorophyll biosynthesis to optimize the greening process in *Arabidopsis*. P Natl Acad Sci USA.

[7] Tominaga-Wada R, Nukumizu Y, Wada T (2013). Flowering is delayed by mutations in homologous genes CAPRICE and TRYPTICHON in the early flowering *Arabidopsis* cpl3 mutant. J Plant Physiol.

[8] Deng L, Qin P, Liu Z, Wang G, Chen W, Tong J (2017). Characterization and fine-mapping of a novel premature leaf senescence mutant yellow leaf and dwarf 1 in rice. Plant Physiol Biochem.

[9] Zheng J, Wu H, Zhu H, Huang C, Liu C, Chang Y (2019). Determining factors, regulation system, and domestication of anthocyanin biosynthesis in rice leaves. New Phytol.

[10] Lonosky PM, Zhang X, Honavar VG, Dobbs DL, Fu A, Rodermel SR (2004). A proteomic analysis of maize chloroplast biogenesis. Plant Physiol.

[11] Hu Y, Chen B (2020). Arbuscular mycorrhiza induced putrescine degradation into gamma-aminobutyric acid, malic acid accumulation, and improvement of nitrogen assimilation in roots of water-stressed maize plants. Mycorrhiza..

[12] Zhou H, Lin-Wang K, Wang F, Espley RV, Ren F, Zhao J (2019). Activator-type R2R3-MYB genes induce a repressor-type R2R3-MYB gene to balance anthocyanin and proanthocyanidin accumulation. New Phytol.

[13] Wu S, Guo Y, Adil MF, Sehar S, Cai B, Xiang Z, et al. Comparative proteomic analysis by iTRAQ reveals that plastid pigment metabolism contributes to leaf color changes in tobacco (*Nicotiana tabacum*) during curing. Int J Mol Sci. 2020;21(7):2394. 10.3390/ijms21072394 PMC7178154 32244294

[14] Shiroshita Y, Yuhazu M, Hase Y, Yamada T, Abe J, Kanazawa A (2020). Characterization of chlorophyll-deficient soybean [*Glycine max* (L.) Merr.] mutants obtained by ion-beam irradiation reveals concomitant reduction in isoflavone levels. Genet Resour Crop Ev.

[15] Li W, Yang S, Lu Z, He Z, Ye Y, Zhao B (2018). Cytological, physiological, and transcriptomic analyses of golden leaf coloration in *Ginkgo biloba* L. Hortic Res.

[16] Gang HX, Liu GF, Chen S, Jiang J. Physiological and transcriptome analysis of a yellow-green leaf mutant in birch (*Betula platyphylla* x B. Pendula). Forests. 2019;10(2):120.

[17] Zhao MH, Li X, Zhang XX, Zhang H, Zhao XY. Mutation mechanism of leaf color in plants: a review. Forests. 2020;11(8):851.

[18] Cedar H, Bergman Y (2012). Programming of DNA methylation patterns. Annu Rev Biochem.

[19] Law JA, Jacobsen SE (2010). Establishing, maintaining and modifying DNA methylation patterns in plants and animals. Nat Rev Genet.

[20] Cao X, Jacobsen SE (2002). Locus-specific control of asymmetric and CpNpG methylation by the DRM and CMT3 methyltransferase genes. P Natl Acad Sci USA.

[21] Jiang SH, Sun QG, Chen M, Wang N, Xu HF, Fang HC (2019). Methylome and transcriptome analyses of apple fruit somatic mutations reveal the difference of red phenotype. BMC Genomics.

[22] Jiang LB, Zhang M, Ma KF. Whole-genome DNA methylation associated with differentially expressed genes regulated anthocyanin biosynthesis within flower color chimera of ornamental tree *Prunus mume*. Forests. 2020;11(1):90.

[23] Xie XB, Li S, Zhang RF, Zhao J, Chen YC, Zhao Q (2012). The bHLH transcription factor MdbHLH3 promotes anthocyanin accumulation and fruit colouration in response to low temperature in apples. Plant Cell Environ.

[24] Nesi N, Jond C, Debeaujon I, Caboche M, Lepiniec L (2001). The *Arabidopsis* TT2 gene encodes an R2R3 MYB domain protein that acts as a key determinant for proanthocyanidin accumulation in developing seed. Plant Cell.

[25] El-Sharkawy I, Liang D, Xu KN (2015). Transcriptome analysis of an apple *(Malus x domestica*) yellow fruit somatic mutation identifies a gene network module highly associated with anthocyanin and epigenetic regulation. J Exp Bot.

[26] Bossdorf O, Arcuri D, Richards CL, Pigliucci M (2010). Experimental alteration of DNA methylation affects the phenotypic plasticity of ecologically relevant traits in *Arabidopsis thaliana*. Evol Ecol.

[27] Chan SW-L, Henderson IR, Zhang X, Shah G, Jacobsen SE (2006). RNAi, DRD1, and histone methylation actively target developmentally important non-CG DNA methylation in *Arabidopsis*. PLoS Genet.

[28] Ausin I, Feng SH, Yu CW, Liu WL, Kuo HY, Jacobsen EL (2016). DNA methylome of the 20-gigabase Norway spruce genome. P Natl Acad Sci USA.

[29] Niederhuth CE, Bewick AJ, Ji L, Alabady MS, Schmitz RJ (2016). Widespread natural variation of DNA methylation within angiosperms. Genome Biol.

[30] Meng D, Dubin M, Zhang P, Osborne EJ, Stegle O, Clark RM (2016). Limited contribution of DNA methylation variation to expression regulation in *Arabidopsis thaliana*. PLoS Genet.

[31] Li X, Zhu J, Hu F, Ge S, Ye M, Xiang H (2012). Single-base resolution maps of cultivated and wild rice methylomes and regulatory roles of DNA methylation in plant gene expression. BMC Genomics.

[32] Xing LB, Li YM, Qi SY, Zhang CG, Ma WC, Zuo XY (2019). Comparative RNA-sequencing and DNA methylation analyses of apple (*Malus domestica* Borkh.) buds with diverse flowering capabilities reveal novel insights into the regulatory mechanisms of flower bud formation. Plant Cell Physiol.

[33] Wu H, Shi N, An X, Liu C, Fu H, Cao L, et al. Candidate genes for yellow leaf color in common wheat (*Triticum aestivum* L.) and major related metabolic pathways according to transcriptome profiling. Int J Mol Sci. 2018;19(6):1564. 10.3390/ijms19061594 PMC6032196 29843474

[34] Song L, Ma Q, Zou Z, Sun K, Yao Y, Tao J (2017). Molecular link between leaf coloration and gene expression of flavonoid and carotenoid biosynthesis in *Camellia sinensis* cultivar ‘Huangjinya’. Front Plant Sci.

[35] Feng L, Gao MJ, Hou RY, Hu XY, Zhang L, Wan XC (2014). Determination of quality constituents in the young leaves of albino tea cultivars. Food Chem.

[36] Tanaka R, Koshino Y, Sawa S, Ishiguro S, Okada K, Tanaka A (2001). Overexpression of chlorophyllide a oxygenase (CAO) enlarges the antenna size of photosystem II in *Arabidopsis thaliana*. Plant J.

[37] Hao N, Du Y, Li H, Wang C, Wang C, Gong S (2018). CsMYB36 is involved in the formation of yellow green peel in cucumber (*Cucumis sativus* L.). Theor Appl Genet.

[38] Maslova TG, Markovskaya EF, Slemnev NN (2020). Functions of carotenoids in leaves of higher plants (an overview). Zh Obshch Biol.

[39] Gu C, Liao L, Zhou H, Wang L, Deng X, Han Y (2015). Constitutive activation of an anthocyanin regulatory gene *PcMYB10.6* is related to red coloration in purple-foliage plum. PLoS One.

[40] Sun W, Meng X, Liang L, Jiang W, Huang Y, He J (2015). Molecular and biochemical analysis of chalcone synthase from freesia hybrid in flavonoid biosynthetic pathway. PLoS One.

[41] Itoh Y, Higeta D, Suzuki A, Yoshida H, Ozeki Y (2002). Excision of transposable elements from the chalcone isomerase and dihydroflavonol 4-reductase genes may contribute to the variegation of the yellow-flowered carnation (*Dianthus caryophyllus*). Plant Cell Physiol.

[42] Arnon DI (1949). Copper enzymes in isolated chloroplasts. Polyphenoloxidase in *Beta vulgaris*. Plant Physiol.

[43] Sun T, Pei T, Yang L, Zhang Z, Li M, Liu Y (2021). Exogenous application of xanthine and uric acid and nucleobase-ascorbate transporter MdNAT7 expression regulate salinity tolerance in apple. BMC Plant Biol.

[44] Akalin A, Kormaksson M, Li S, Garrett-Bakelman FE, Figueroa ME, Melnick A (2012). methylKit: a comprehensive R package for the analysis of genome-wide DNA methylation profiles. Genome Biol.

[45] Boyle EI, Weng S, Gollub J, Jin H, Botstein D, Cherry JM, Sherlock G (2004). GO::TermFinder-open source software for accessing gene ontology information and finding significantly enriched gene ontology terms associated with a list of genes. Bioinformatics..

[46] Kanehisa M, Araki M, Goto S, Hattori M, Hirakawa M, Itoh M (2008). KEGG for linking genomes to life and the environment. Nucleic Acids Res.

[47] Chen S, Zhou Y, Chen Y, Gu J (2018). Fastp: an ultra-fast all-in-one FASTQ preprocessor. Bioinformatics..

[48] Langmead B, Trapnell C, Pop M, Salzberg SL (2009). Ultrafast and memory-efficient alignment of short DNA sequences to the human genome. Genome Biol.

[49] Kim D, Langmead B, Salzberg SL (2015). HISAT: a fast spliced aligner with low memory requirements. Nat Methods.

[50] Li B, Dewey CN (2011). RSEM: accurate transcript quantification from RNA-Seq data with or without a reference genome. BMC Bioinformatics.

[51] Love MI, Huber W, Anders S (2014). Moderated estimation of fold change and dispersion for RNA-seq data with DESeq2. Genome Biol.

[52] Tarazona S, Garcia-Alcalde F, Dopazo J, Ferrer A, Conesa A (2011). Differential expression in RNA-seq: a matter of depth. Genome Res.

[53] Bowen J, Ireland HS, Crowhurst R, Luo ZW, Watson AE, Foster T (2014). Selection of low-variance expressed *Malus x domestica* (apple) genes for use as quantitative PCR reference genes (housekeepers). Tree Genet Genomes.

[54] Livak KJ, Schmittgen TD (2001). Analysis of relative gene expression data using real-time quantitative PCR and the 2^-ΔΔCT^ method. Methods..

