# Supplementary material for: Methylome and transcriptome analyses of three different degrees of albinism in apple seedlings
Source: BMC Genomics. 2022 Apr 19;23:310. doi: 10.1186/s12864-022-08535-3 (PMC9016989; doi:10.1186/s12864-022-08535-3)
Supplement: Supplementary file 1 — Additional file 1: Table S1. Information on the bisulfite sequencing libraries. Table S2. The coverage of the intergenic region. Table S3. The DMRs distributed on each chromosome. Table S4. RNA-seq sequencing data and mapping of the genome. Table S5. Differentially expressed genes involved in the flavonoid, chlorophyll and carotenoid biosynthesis pathways in WM/NL, PM/NL and LM/NL. Table S6. DMR-associated DEGs involved in the carotenoid and flavonoid biosynthesis pathways. Table S7. The average methylation levels in NL, WM, PM and LM. Table S8. The primers used in qRT-PCR and BS-PCR. Fig. S1. Information of the sequencing and mapping. Fig. S2. The global methylome in NL, WM, PM and LM. Fig. S3. The chromosomal mCs distributions in NL, WM, PM and LM. Fig. S4. The KEGG analysis of DMRs in WM/NL, PM/NL and LM/NL. Fig. S5. The principal component analysis (PCA) of expressed genes. Fig. S6. DGEs in WM/NL, PM/NL and LM/NL. Fig. S7. GO and KEGG pathway analyses of the DEGs in WM/NL, PM/NL and LM/NL. Fig. S8. Number of differentially methylated genes of CG, CHG and CHH between DMRs and DEGs in WM/NL, PM/NL and LM/NL. Fig. S9. Relationship between differential methylation and gene expression. Fig. S10. DNA methylation validated in WM/NL, PM/NL and LM/NL. Fig. S11. Gene expression validation involved in the chlorophyll, carotenoid and flavonoid biosynthesis pathways. [file 12864_2022_8535_MOESM1_ESM.docx]

**Table S1.** Information on the bisulfite sequencing libraries.

| Sample | Clean Reads number | Mapped Reads number | Mapping Rate (%) | Bisulfite Conversion Rate (%) |
| --- | --- | --- | --- | --- |
| NL | 103226694 | 100699326 | 97.55% | 99.29 |
| WM | 99349754 | 97804686 | 98.44% | 99.28 |
| PM | 103548598 | 102348440 | 98.84% | 99.19 |
| LM | 105546088 | 104361960 | 98.88% | 99.25 |

NL, WM, PM and LM represent normal leaves, white-leaves mutant, piebald-leaves mutant and light-green-leaves mutant of apple, respectively. Bisulfite Conversion Rate =1-methylation rate of Lambda DNA

**Table S2.** The coverage of the intergenic region.

| Location | C | | | |  | CG | | | |
| --- | --- | --- | --- | --- | --- | --- | --- | --- | --- |
|  | NL | WM | LM | LM |  | NL | WM | LM | LM |
| Genebody | 89.09 | 84.78 | 88.74 | 88.32 |  | 89.09 | 88.72 | 84.4 | 88.37 |
| Upstream-2k | 82.79 | 79.37 | 82.17 | 81.48 |  | 83.37 | 82.69 | 80.12 | 82.36 |
| Downstream-2k | 84.96 | 81.03 | 84.37 | 83.84 |  | 85.16 | 84.59 | 81.29 | 84.24 |
| 5-UTR | 80.64 | 71.33 | 80.34 | 77.51 |  | 84.87 | 84.22 | 76.17 | 82.4 |
| 3-UTR | 91.98 | 88.18 | 91.58 | 91.42 |  | 91.89 | 91.51 | 88.24 | 91.42 |
| Exon | 91.50 | 85.99 | 91.28 | 90.36 |  | 92.06 | 91.8 | 86.11 | 90.98 |
| CDS | 93.02 | 87.74 | 92.83 | 92.06 |  | 93.04 | 92.85 | 87.08 | 92.04 |
| Intron | 86.37 | 83.41 | 85.88 | 86.02 |  | 84.19 | 83.63 | 81.57 | 84.06 |

| Location | CHG | | | |  | CHH | | | |
| --- | --- | --- | --- | --- | --- | --- | --- | --- | --- |
|  | NL | WM | LM | LM |  | NL | WM | LM | LM |
| Genebody | 90.97 | 90.67 | 87.13 | 90.42 |  | 88.63 | 88.27 | 84.27 | 87.8 |
| Upstream-2k | 83.55 | 83 | 80.17 | 82.56 |  | 82.56 | 81.94 | 79.12 | 81.15 |
| Downstream-2k | 85.92 | 85.39 | 81.99 | 85.03 |  | 84.74 | 84.14 | 80.81 | 83.56 |
| 5-UTR | 85.1 | 84.6 | 76.91 | 82.72 |  | 78.91 | 78.71 | 69.25 | 75.5 |
| 3-UTR | 92.64 | 92.32 | 89.11 | 92.21 |  | 91.84 | 91.41 | 87.94 | 91.23 |
| Exon | 93.29 | 93.07 | 88.54 | 92.46 |  | 90.86 | 90.64 | 85.22 | 89.62 |
| CDS | 94.33 | 94.16 | 89.78 | 93.61 |  | 92.61 | 92.42 | 87.27 | 91.58 |
| Intron | 87.65 | 87.21 | 85.1 | 87.51 |  | 86.4 | 85.91 | 83.32 | 85.99 |

**Table S3.** The DMRs distributed on each chromosome.

| Chromosome  (WM/NL) | Numbers of DMR | Length of DMR region | Numbers of DMR | Length of DMR region | Numbers of DMR | Length of DMR region |
| --- | --- | --- | --- | --- | --- | --- |
|  | CG | | CHG | | CHH | |
| Chr1 | 785 | 157000 | 757 | 151400 | 1304 | 260800 |
| Chr2 | 1001 | 200200 | 1065 | 213000 | 1756 | 351200 |
| Chr3 | 947 | 189400 | 1180 | 236000 | 1691 | 338200 |
| Chr4 | 749 | 149800 | 827 | 165400 | 1449 | 289800 |
| Chr5 | 1106 | 221200 | 1330 | 266000 | 1953 | 390600 |
| Chr6 | 788 | 157600 | 859 | 171800 | 1434 | 286800 |
| Chr7 | 851 | 170200 | 926 | 185200 | 1479 | 295800 |
| Chr8 | 760 | 152000 | 823 | 164600 | 1271 | 254200 |
| Chr9 | 939 | 187800 | 1041 | 208200 | 1647 | 329400 |
| Chr10 | 1053 | 210600 | 1173 | 234600 | 1876 | 375200 |
| Chr11 | 1065 | 213000 | 1088 | 217600 | 1805 | 361000 |
| Chr12 | 889 | 177800 | 1028 | 205600 | 1592 | 318400 |
| Chr13 | 917 | 183400 | 1126 | 225200 | 1727 | 345400 |
| Chr14 | 790 | 158000 | 869 | 173800 | 1376 | 275200 |
| Chr15 | 1415 | 283000 | 1476 | 295200 | 2465 | 493000 |
| Chr16 | 912 | 182400 | 1078 | 215600 | 1723 | 344600 |
| Chr17 | 866 | 173200 | 1034 | 206800 | 1694 | 338800 |
| Total | 15833 | 3166600 | 17680 | 3536000 | 28242 | 5648400 |

| Chromosome  (PM/NL) | Numbers of DMR | Length of DMR region | Numbers of DMR | Length of DMR region | Numbers of DMR | Length of DMR region |
| --- | --- | --- | --- | --- | --- | --- |
|  | CG | | CHG | | CHH | |
| Chr1 | 847 | 169400 | 910 | 182000 | 1719 | 343800 |
| Chr2 | 1265 | 253000 | 1314 | 262800 | 2452 | 490400 |
| Chr3 | 1189 | 237800 | 1363 | 272600 | 2435 | 487000 |
| Chr4 | 909 | 181800 | 1043 | 208600 | 1991 | 398200 |
| Chr5 | 1398 | 279600 | 1654 | 330800 | 2810 | 562000 |
| Chr6 | 986 | 197200 | 1033 | 206600 | 2024 | 404800 |
| Chr7 | 1039 | 207800 | 1121 | 224200 | 2108 | 421600 |
| Chr8 | 911 | 182200 | 997 | 199400 | 1767 | 353400 |
| Chr9 | 1054 | 210800 | 1207 | 241400 | 2176 | 435200 |
| Chr10 | 1313 | 262600 | 1450 | 290000 | 2691 | 538200 |
| Chr11 | 1282 | 256400 | 1298 | 259600 | 2508 | 501600 |
| Chr12 | 1096 | 219200 | 1203 | 240600 | 2149 | 429800 |
| Chr13 | 1130 | 226000 | 1286 | 257200 | 2449 | 489800 |
| Chr14 | 981 | 196200 | 1062 | 212400 | 2009 | 401800 |
| Chr15 | 1784 | 356800 | 1761 | 352200 | 3434 | 686800 |
| Chr16 | 1000 | 200000 | 1284 | 256800 | 2364 | 472800 |
| Chr17 | 1046 | 209200 | 1157 | 231400 | 2365 | 473000 |
| Total | 19230 | 3846000 | 21143 | 4228600 | 39451 | 7890200 |

| Chromosome  (LM/NL) | Numbers of DMR | Length of DMR region | Numbers of DMR | Length of DMR region | Numbers of DMR | Length of DMR region |
| --- | --- | --- | --- | --- | --- | --- |
|  | CG | | CHG | | CHH | |
| Chr1 | 863 | 172600 | 921 | 184200 | 1534 | 306800 |
| Chr2 | 1258 | 251600 | 1305 | 261000 | 1998 | 399600 |
| Chr3 | 1138 | 227600 | 1450 | 290000 | 2100 | 420000 |
| Chr4 | 949 | 189800 | 1112 | 222400 | 1701 | 340200 |
| Chr5 | 1360 | 272000 | 1665 | 333000 | 2269 | 453800 |
| Chr6 | 928 | 185600 | 1117 | 223400 | 1708 | 341600 |
| Chr7 | 1065 | 213000 | 1203 | 240600 | 1835 | 367000 |
| Chr8 | 920 | 184000 | 995 | 199000 | 1544 | 308800 |
| Chr9 | 1074 | 214800 | 1270 | 254000 | 1954 | 390800 |
| Chr10 | 1384 | 276800 | 1511 | 302200 | 2356 | 471200 |
| Chr11 | 1269 | 253800 | 1302 | 260400 | 2104 | 420800 |
| Chr12 | 1123 | 224600 | 1223 | 244600 | 1857 | 371400 |
| Chr13 | 1091 | 218200 | 1398 | 279600 | 2071 | 414200 |
| Chr14 | 986 | 197200 | 1058 | 211600 | 1706 | 341200 |
| Chr15 | 1728 | 345600 | 1856 | 371200 | 2862 | 572400 |
| Chr16 | 1168 | 233600 | 1296 | 259200 | 2091 | 418200 |
| Chr17 | 1102 | 220400 | 1187 | 237400 | 1934 | 386800 |
| Total | 19406 | 3881200 | 21869 | 4373800 | 33624 | 6724800 |

**Table S4.** RNA-seq sequencing data and mapping of the genome.

| Sample | Raw Reads | Clean Reads | Mapped Reads |
| --- | --- | --- | --- |
| NL-1 | 57221284 | 57145600 (99.87%) | 52330037 (92.34%) |
| NL-2 | 49684638 | 49620774 (99.87%) | 45760445 (92.78%) |
| NL-3 | 65367556 | 65286760 (99.88%) | 59595608 (92.27%) |
| WM-1 | 54162256 | 57578948 (99.89%) | 49728599 (92.94%) |
| WM-2 | 42135284 | 65165456 (99.88%) | 39079903 (93.70%) |
| WM-3 | 51190134 | 49170284 (99.89%) | 47213627 (93.03%) |
| PM-1 | 54312306 | 54243918 (99.87%) | 50157491 (93.14%) |
| PM-2 | 53614804 | 53549778 (99.88%) | 49793308 (93.48%) |
| PM-3 | 40133282 | 40090682 (99.89%) | 36819129 (93.10%) |
| LM-1 | 57643490 | 54098354 (99.88%) | 53289513 (93.15%) |
| LM-2 | 65245626 | 42098640 (99.91%) | 59789912 (92.71%) |
| LM-3 | 49223464 | 51131648 (99.89%) | 45134477 (92.59%) |

NL, WM, PM and LM represent normal leaves, white-leaves mutant, piebald-leaves mutant and light-green-leaves mutant of apple, respectively.

**Table S5.** Differentially expressed genes involved in the flavonoid, chlorophyll and carotenoid biosynthesis pathways in WM/NL, PM/NL, and LM/NL.

| Function | Term | Annoation | WM/NL | PM/NL | LM/NL |
| --- | --- | --- | --- | --- | --- |
| Flavanone biosynthesis | PAL | Phenylalanine ammonia-lyase | MD01G1106900  MD04G1096200  MD12G1116700 | MD01G1106900  MD04G1096200  MD12G1116700 | MD01G1106900  MD04G1096200  MD12G1116700 |
|  | 4CL | 4-coumarate: CoA ligase | MD13G1257800 | MD13G1257800 | MD13G1257800 |
|  | CHS | Chalcone isomerase | MD04G1003000 | MD04G1003000 | MD04G1003000 |
|  | CHI | chalcone-flavonone isomerase | MD01G1167300  MD07G1186300  MD07G1233400 | MD01G1167300  MD07G1186300  MD07G1233400 | MD01G1167300  MD07G1186300  MD07G1233400 |
|  | DFR | bifunctional dihydroflavonol 4-reductase/flavanone 4-reductase | MD08G1028600  MD08G1191700  MD15G1024100 | MD08G1028600  MD08G1191700  MD15G1024100 | MD08G1028600  MD08G1191700  MD15G1024100 |
|  | LAR | leucoanthocyanidin reductase | MD06G1211400  MD13G1046900  MD16G1048500 | MD06G1211400  MD13G1046900  MD16G1048500 | MD06G1211400  MD13G1046900  MD16G1048500 |
|  | ANS | leucoanthocyanidin dioxygenase | MD03G1001100  MD06G1071600  MD07G1222600 | MD03G1001100  MD06G1071600  MD07G1222600 | MD03G1001100  MD06G1071600  MD07G1222600 |
| Chiorophyll biosynthesis | HEMA | Glutamyl-tRNA reductase | MD08G1039900 | MD08G1039900 | MD08G1039900 |
|  | CAO | chlorophyllide an oxygenase | MD08G1162200 | MD08G1162200 | MD08G1162200 |
|  | CRD1 | Mg-protoporphyrin IX monomethylester | MD00G1107700 | MD00G1107700 | MD00G1107700 |
| Carotenoid biosythesis | PDS | Phytonene desaturase | MD15G1038500 | MD15G1038500 | MD15G1038500 |
|  | ZDS | ζ-carotene desaturase | MD12G1237300 | MD12G1237300 | MD12G1237300 |
|  | NCED | 9-cis-epoxycarotenoid dioxygenase | MD05G1207300 | MD05G1282700 | MD05G1282700 |

**Table S6.** DMR-associated DEGs involved in the carotenoid and flavonoid biosynthesis pathways. meth.diff = methylation difference; p-value = the p-value of methylation difference; log2(FC) = differential multiples of gene expression; P-Value = P values of different genes; position = The relationship between DMR and gene location; Methylation type = The type of methylation.

|  | Type | Annoation | Gene ID | meth.diff | | p-value | log2(FC) | P-Value | position | Methylation type |
| --- | --- | --- | --- | --- | --- | --- | --- | --- | --- | --- |
| WM/NL | CG | CHI | MD07G1186300 | 27.50 | 1.46E-04 | | -1.54 | 5.94E-27 | Genebody | Hyper- |
|  |  | CHI | MD07G1233400 | 25.15 | 4.47E-04 | | -5.43 | 1.04E-169 | Genebody | Hyper- |
|  | CHG | CHI | MD07G1186300 | 26.79 | 5.74E-04 | | -1.54 | 5.94E-27 | Genebody | Hyper- |
|  |  | CHI | MD07G1233400 | 28.29 | 5.01E-04 | | -5.43 | 1.04E-169 | Genebody | Hyper- |
|  | CHH | CHS | MD04G1003000 | 24.94 | 3.04E-12 | | -2.97 | 6.24E-32 | Promoter | Hyper- |
|  |  | CHI | MD07G1233400 | 15.94 | 1.36E-05 | | -5.43 | 1.04E-169 | Genebody | Hyper- |
| PM/NL | CG | PDS | MD15G1038500 | 29.90 | 6.33E-04 | | -1.22 | 1.24E-02 | Promoter | Hyper- |
|  |  | DFR | MD15G1024100 | 34.57 | 1.40E-06 | | -1.59 | 2.34E-48 | Genebody | Hyper- |
|  | CHG | PAL | MD04G1096200 | 27.17 | 5.43E-04 | | -1.59 | 5.63E-49 | Genebody | Hyper- |
|  |  | CHI | MD07G1186300 | 26.32 | 4.34E-05 | | -2.01 | 7.54E-71 | Genebody | Hyper- |
|  |  | CHI | MD07G1233400 | 26.46 | 1.04E-04 | | -2.12 | 2.23E-43 | Promoter | Hyper- |
|  |  | CHI | MD01G1167300 | 43.57 | 2.34E-06 | | -1.85 | 1.58E-35 | Genebody | Hyper- |
|  |  | DFR | MD15G1024100 | 46.36 | 5.47E-09 | | -1.59 | 2.34E-48 | Genebody | Hyper- |
|  | CHH | CHS | MD04G1003000 | 23.46 | 3.19E-05 | | -1.54 | 6.61E-28 | Promoter | Hyper- |
|  |  | CHI | MD07G1186300 | 23.70 | 3.49E-06 | | -2.01 | 7.54E-71 | Promoter | Hyper- |
|  |  | LAR | MD16G1048500 | 15.82 | 6.91E-05 | | -2.22 | 5.88E-37 | Genebody | Hyper- |
|  |  | ANS | MD03G1001100 | 19.05 | 2.36E-13 | | -1.94 | 2.17E-75 | Promoter | Hyper- |
|  |  | DFR | MD15G1024100 | 18.13 | 2.87E-07 | | -1.59 | 2.34E-48 | Promoter | Hyper- |
| LM/NL | CG | CHS | MD04G1003000 | 30.15 | 9.44E-04 | | -1.29 | 4.15E-54 | Promoter | Hyper- |
|  |  | CHI | MD07G1186300 | 34.24 | 6.05E-06 | | -1.41 | 1.19E-59 | Genebody | Hyper- |
|  |  | CHI | MD07G1233400 | 37.81 | 1.90E-06 | | -1.65 | 1.19E-105 | Genebody | Hyper- |
|  | CHG | CHS | MD04G1003000 | 38.69 | 6.04E-05 | | -1.29 | 4.15E-54 | Promoter | Hyper- |
|  |  | CHI | MD07G1186300 | 26.69 | 8.56E-08 | | -1.41 | 1.19E-59 | Genebody | Hyper- |
|  | CHH | LAR | MD16G1048500 | 16.70 | 2.56E-04 | | -2.46 | 1.20E-91 | Genebody | Hyper- |

**Table S7.** The average methylation level in NL, WM, PM and LM.

| Varieties | C | CG | CHG | CHH |
| --- | --- | --- | --- | --- |
| NL | 19.17 | 62.85 | 44.52 | 7.31 |
| WM | 19.45 | 62.93 | 44.56 | 7.65 |
| PM | 21.59 | 67.00 | 49.25 | 9.05 |
| LM | 18.96 | 63.50 | 44.92 | 6.73 |

**Table S8.** The primers used in qRT-PCR and BS-PCR.

|  | Gene | | Annotation | | | Forward | Reverse | |
| --- | --- | --- | --- | --- | --- | --- | --- | --- |
| qRT-PCR | | MD04G1096200 | | PAL | CTTCCACATTTTTGGTTGCGCT | | | GTCCTCTTTGCTACTTGGCTCA |
|  |  | MD12G1116700 | | PAL | TGTTGGTTCTGGCTTGGCTTCC | | | AGGGTGGTGCTTCAACTTGTG |
|  |  | MD04G1003000 | | CHS | GGACTGGAACTCACTCTT | | | TACCGTAATCCGACAACA |
|  |  | MD01G1167300 | | CHI | CTTCTTTGATGCTCTTATTTCTG | | | CTCCACTTTGAGTTTCGACTCTTATC |
|  |  | MD07G1186300 | | CHI | GGCTCCACCACCATCGCTCGCT | | | CCCCTGAATCTCCAACCCCCTC |
|  |  | MD07G1233400 | | CHI | ATGCTCTTGTTTCTGCTCCTGT | | | TGATCTTTGACTCTTCCTTCCC |
|  |  | MD08G1191700 | | HEMA | CACTCGCATTCCATCGCTCCTC | | | CTCCTTGGCTTCCTTGGCTTCC |
|  |  | MD15G1024100 | | DFR | CATCGGCTCTTGGCTCGTCATG | | | ACAGCGTCAGGTGCGTCTCC |
|  |  | MD03G1001100 | | ANS | GAGTGCAGGGCCTGCATTGTT | | | ATTTTTGGAGCTGGCTTTCGA |
|  |  | MD08G1207100 | | HEMA1 | GCCGCTTCTTCTGATGCTTCTACC | | | TCCGATGACCACAATGCTGCTTC |
|  |  | MD14G1147900 | | CHLM | GATTGCGCATCTTGCCTCATTG | | | AGCCCTCTCCACATCTGCCTCT |
|  |  | MD05G1207300 | | NCED | GTTCACTTATTTGGCTCTCGCG | | | TATCCATCGTCCTCATTCTCCG |
|  |  | MD13G1004100 | | HEMC | AGCAACAGACCCAGAAGTCCAAAC | | | TTGTCTCGTAAGCCTGAGCAAGTG |
|  |  | MD16G1048500 | | LAR | GTGGACGACATGCGAACAATCAAC | | | TCTTCAGTGACGGTGACTCTAGGG |
| BS-PCR | | MD13G1257800 | | 4CL | TAAAATAGTAGGTGTTAAAAATTG | | | ACCTACTACAAATACTTAATCATAAACATA |
|  |  | MD04G1003000 | | CHS | TAGGAATTATAAAGGTGTGGTGAT  TAGGAATTATAAAGGTGTGGTGAT | | | ATCAATACATACATACAAAAATATTCAACA  AAATCAATACATACATACAAAAATATTCAA |
|  |  | MD07G1233400 | | CHI | TTTATTTTTTTGTAATTTTTAATATTGTTG | | | ATCAACTCACTTAATCAACTCAACC |
|  |  | MD15G1024100 | | DFR | TTTGAAGGTATGTTAGAAAGTAAAAA  GAGTAGGTAATAAATGAAGTTAAGAAT | | | AAATAAACATACTAACCCTACTTAATAAT  ATTTATCTCAACTTCCAAAACATCA |
|  |  | MD03G1001100 | | ANS | TTATATAAAGTGGAAAATTAGGAAT | | | AATTAATTCCACTTAAATCTACAAAAC |
|  |  | MD15G1038500 | | PDS | GTAGGTGGTTTTTATAGGAAAGA  TTTTTAAGAAAAAGGAAAAGTAGTAT | | | CACCATTAAAAACCCAAAACC  ATTAAAAACCCAAAACCACA |





**Fig. S1.** Information of the sequencing and mapping. (A) Cumulative coverage of corresponding depth and coverage of corresponding depth in four apple leaves. The y axis indicates proportion of C bases in the genome is not less than the depth of the sequence, which accounts for the proportion of all C bases in the genome; x axis indicates read depth. (B) Depth distribution of DNA sequencing. The y axis indicates fraction of target bases. (C) The percentage of methylcytosines identified for each sequence context from four apple leaves.


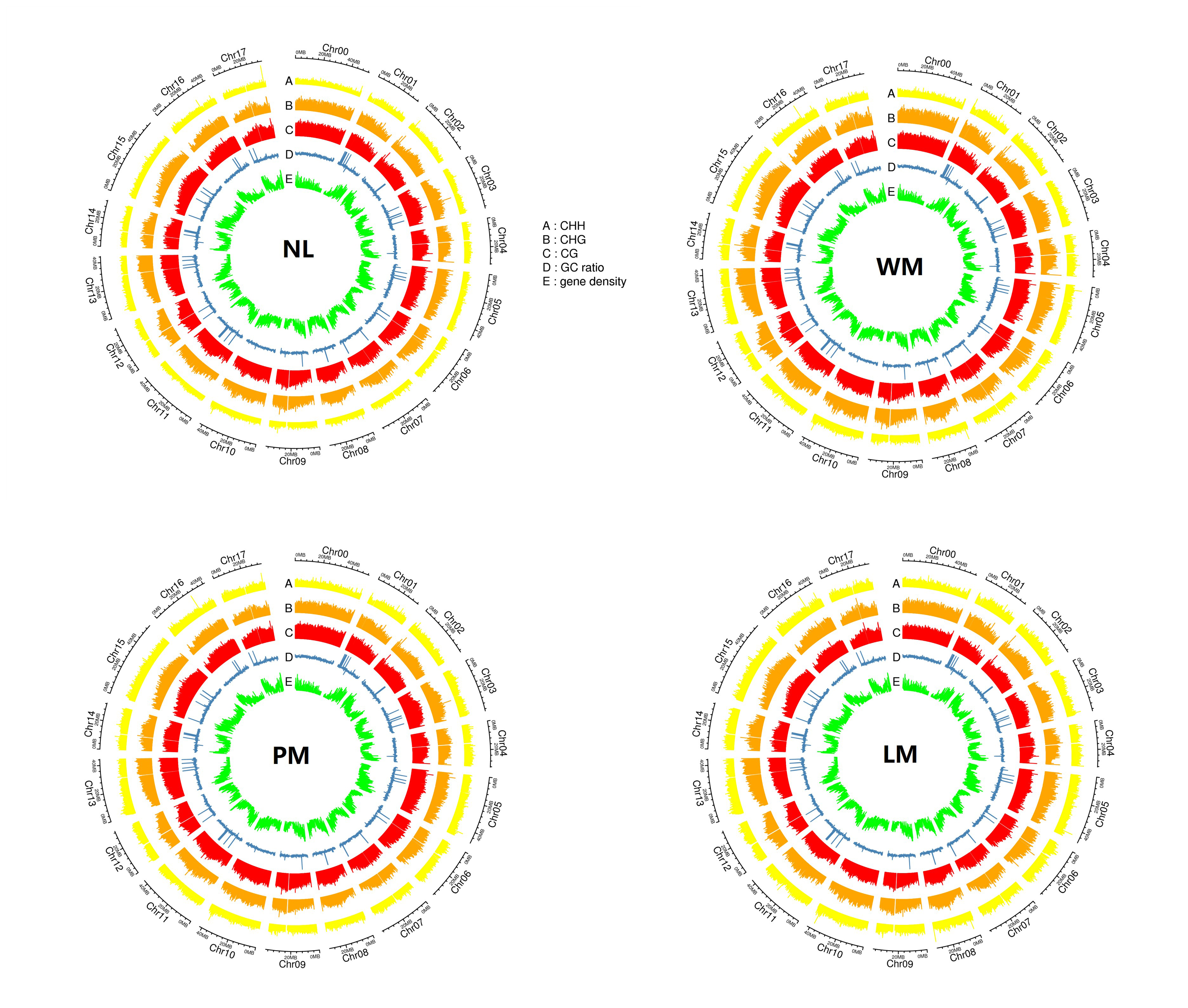


**Fig. S2** The global methylome in NL, WM, PM, and LM. Density plots of mCs in different sequence contexts (A, CHH; B, CHG and C, CG), CG ratio (D), and gene density (E) are shown in the maps. Chromosome name is indicated on the outer rim.


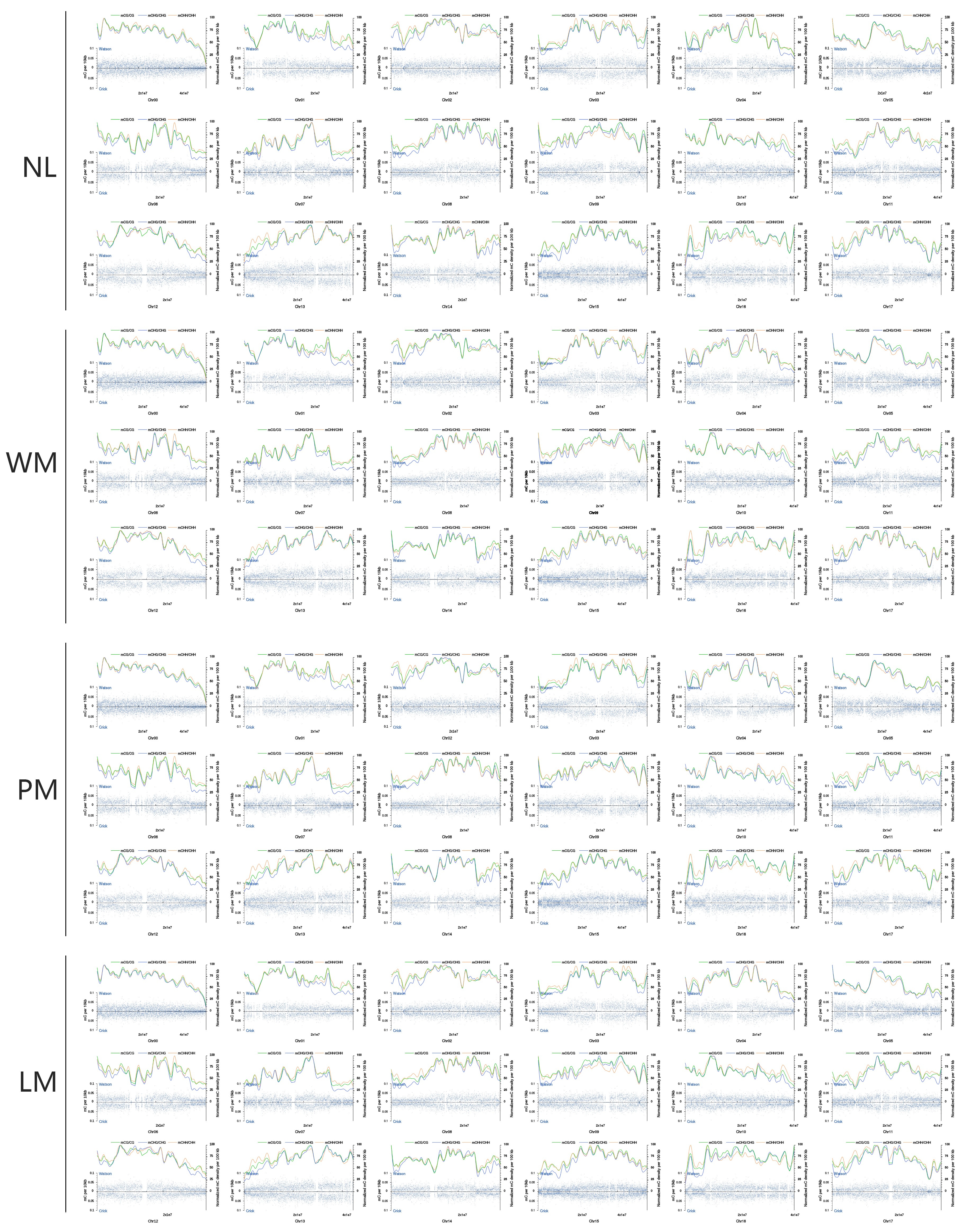


**Fig. S3.** The chromosomal mCs distributions in NL, WM, PM, and LM. The horizontal axis represents the chromosome, from left to right indicate the starting and ending point of the chromosome. The vertical axis on the left shows the mC density calculated for the window of 10 kb, the blue dot indicates the distribution of mC density on the chromosome. The lines represent mC densities in CG (green), CHG (blue), and CHH (orange) contexts, respectively.


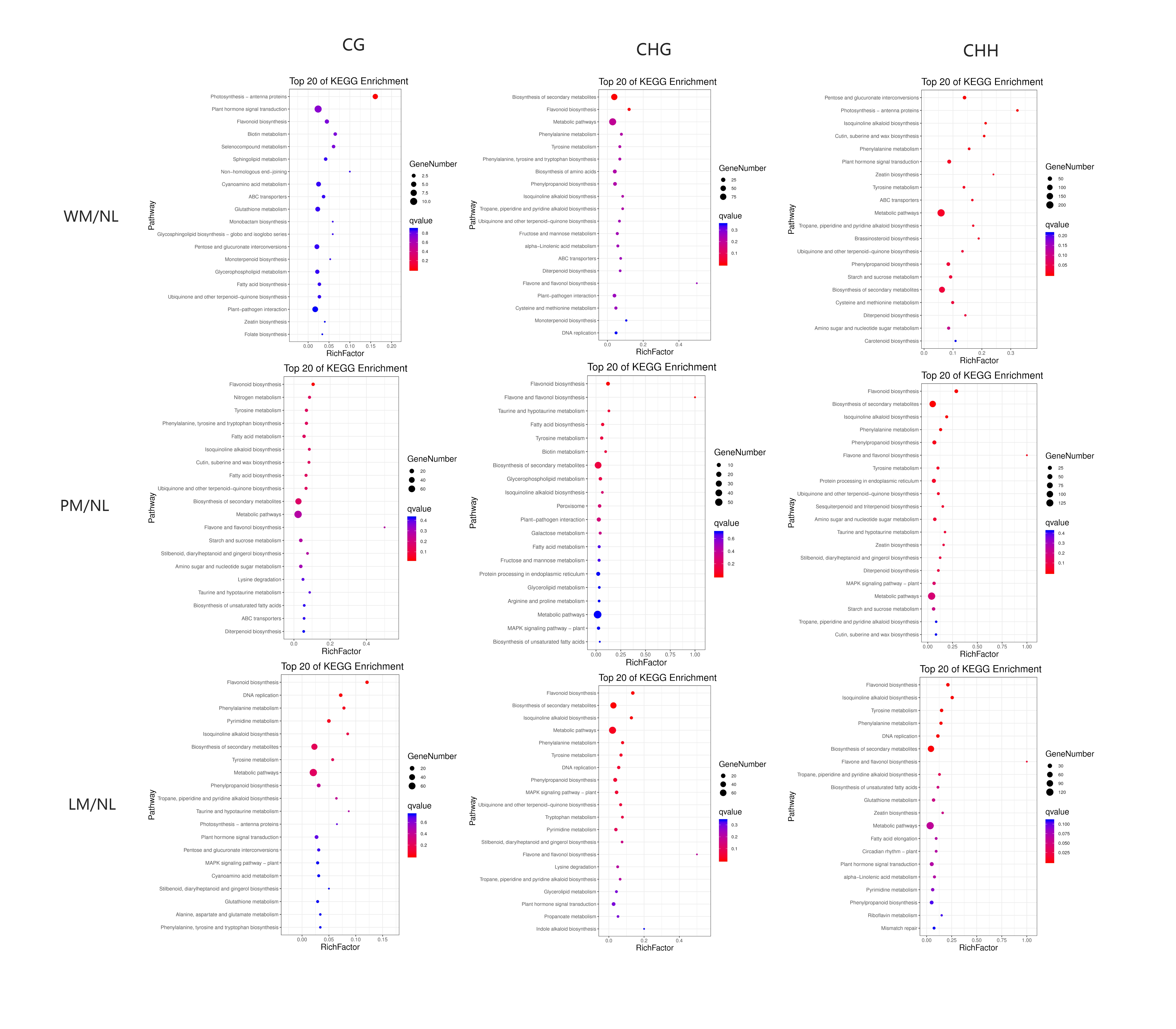


**Fig. S4.** The KEGG analysis of DMRs in WM/NL, PM/NL, and LM/NL. Differential methylation region in CG and CHG, and CHH were analyzed in WM/NL, PM/NL and LM/NL, respectively. Size of the circle represents gene numbers and the blue color represents the q-value.





**Fig. S5** The principal component analysis (PCA) of expressed genes. **
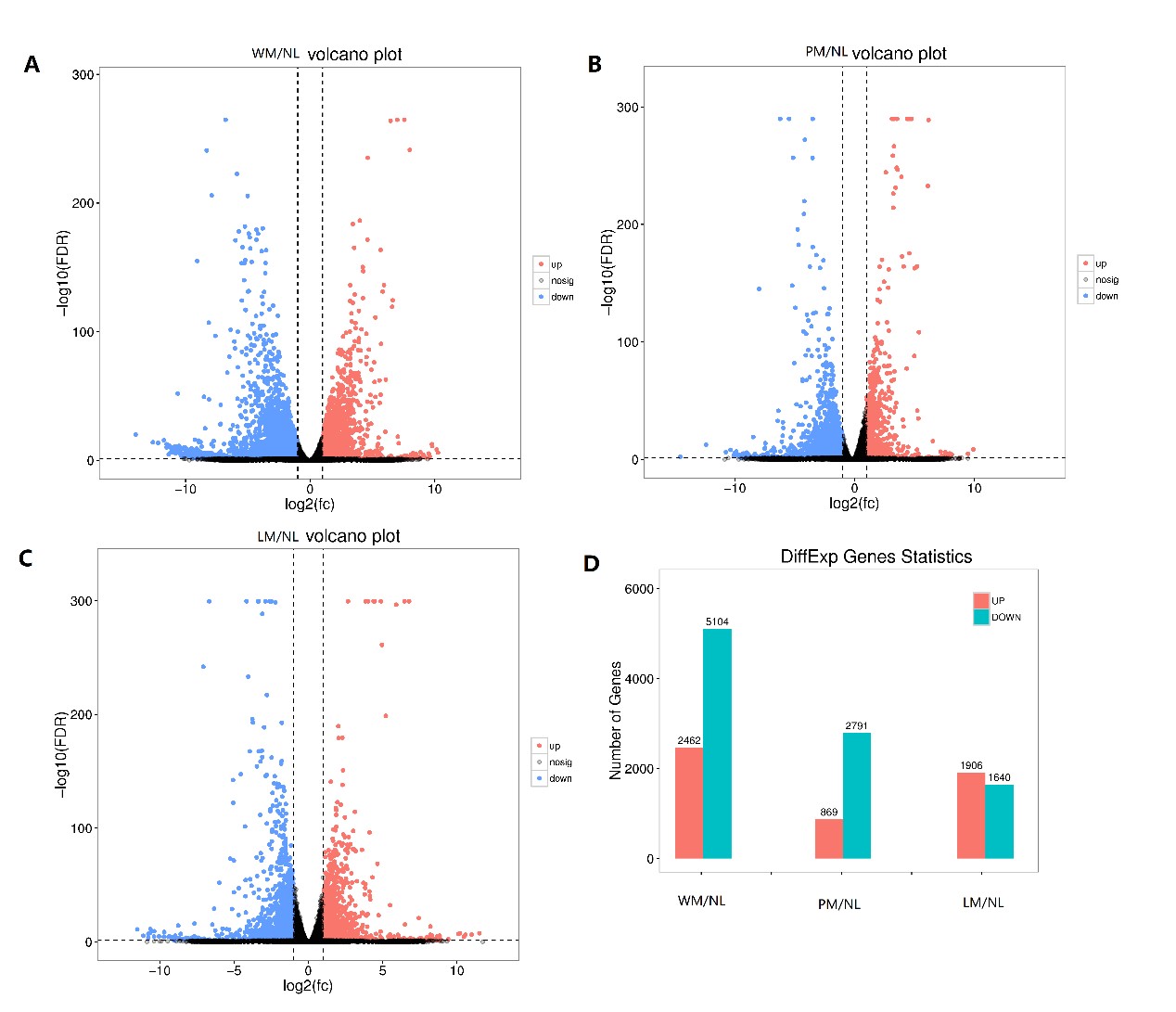
**

**Fig. S6.** DEGs in WM/NL, PM/NL, and LM/NL. Volcano plot of differential gene expression in three comparisons: WM/NL (a), PM/NL (b) and LM/NL (c). Orange spots indicate up-regulated genes and the blue indicate down-regulated genes. (d) Numbers of differentially expressed genes (DEGs) in three comparisons.


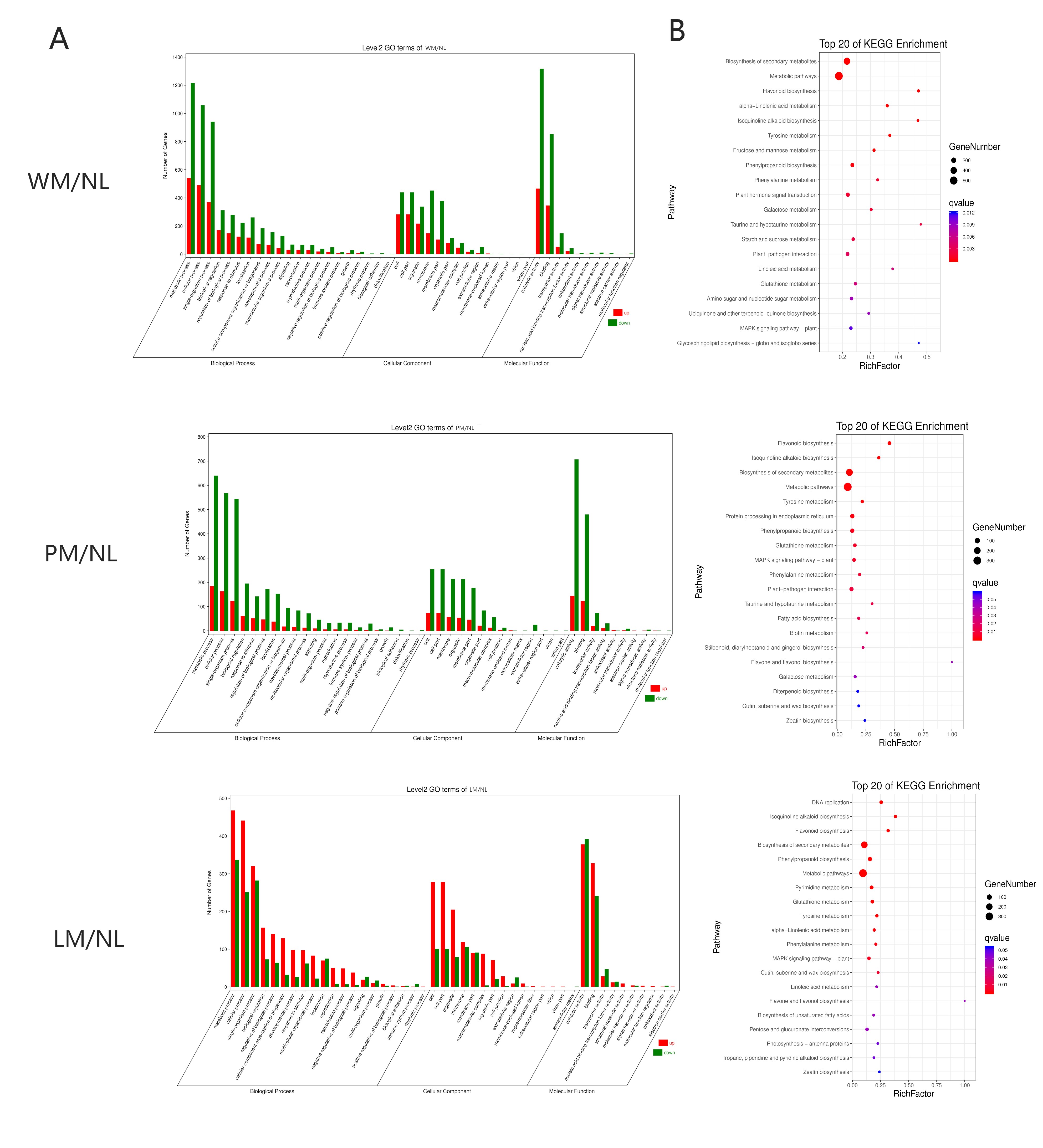


**Fig. S7.** GO and KEGG pathway analyses of the DEGs in WM/NL, PM/NL, and LM/NL. (a) Go analyses for WM/NL, PM/NL and LM/NL comparisons. (b) KEGG pathway analyses for WM/NL, PM/NL and LM/NL comparisons.


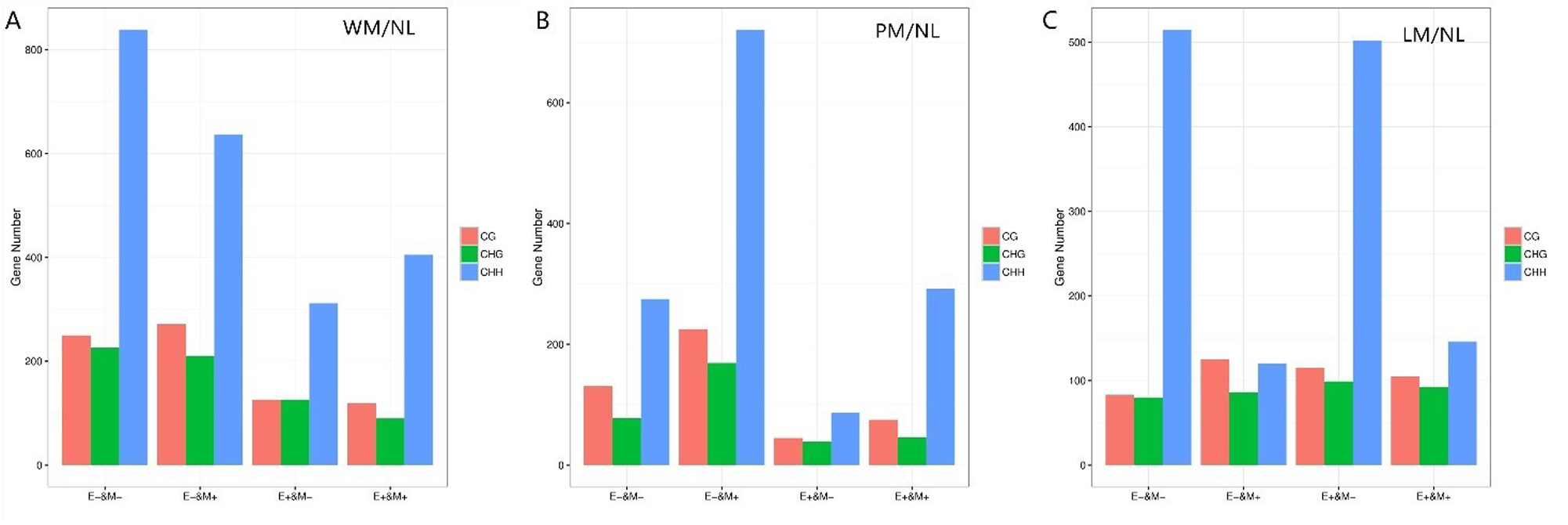


**Fig. S8** Number of differentially methylated genes of CG, CHG, and CHH between DMRs and DEGs in WM/NL, PM/NL and LM/NL. The y axis indicates the different trend of genes, the y axis indicates the number of genes, and different colors represent different types of methylation. E+/E- in the x axis represents the increased/decreased expression of DEGs. M+/M- in the x axis denotes the up-regulated/down-regulated methylation of DMRs.


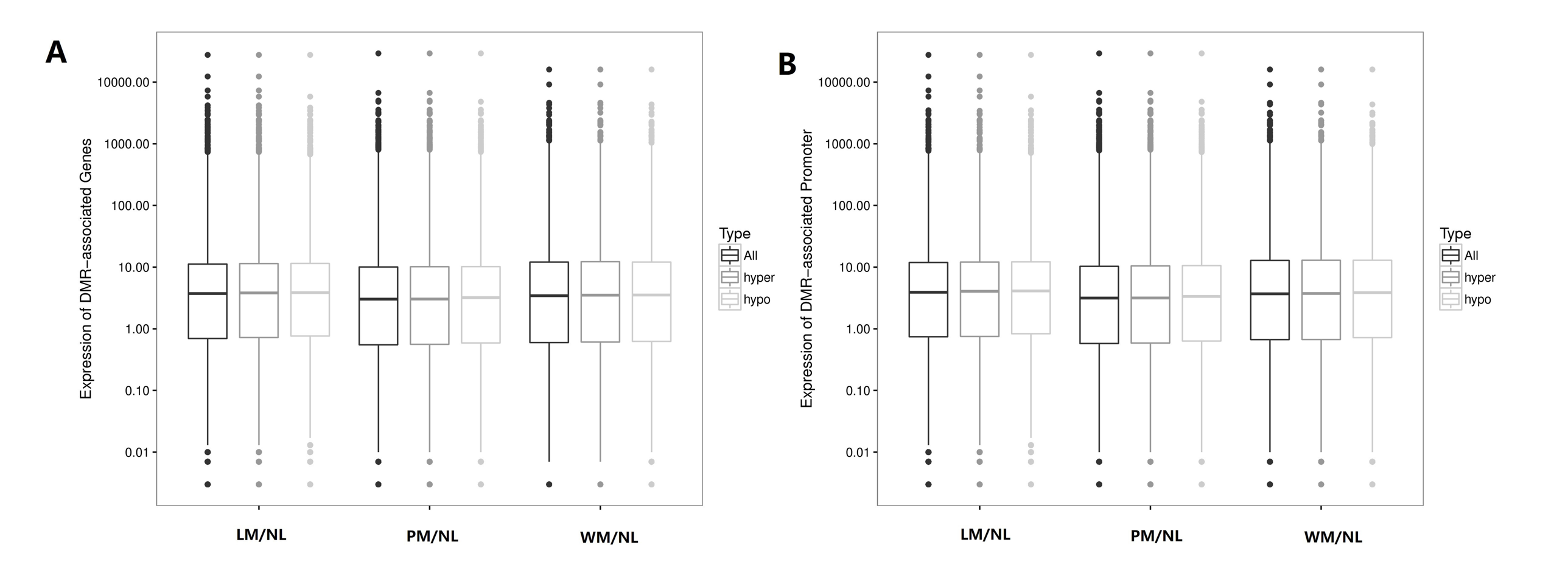


**Fig. S9** Relationship between differential methylation and gene expression. (A) The boxplots of the differential expression of all genes and genes associated with DMRs. (B) The boxplots of the differential expression of all genes and promoters associated with DMRs. Boxes indicate the quartiles; the dotted line represent the date from the min to max.


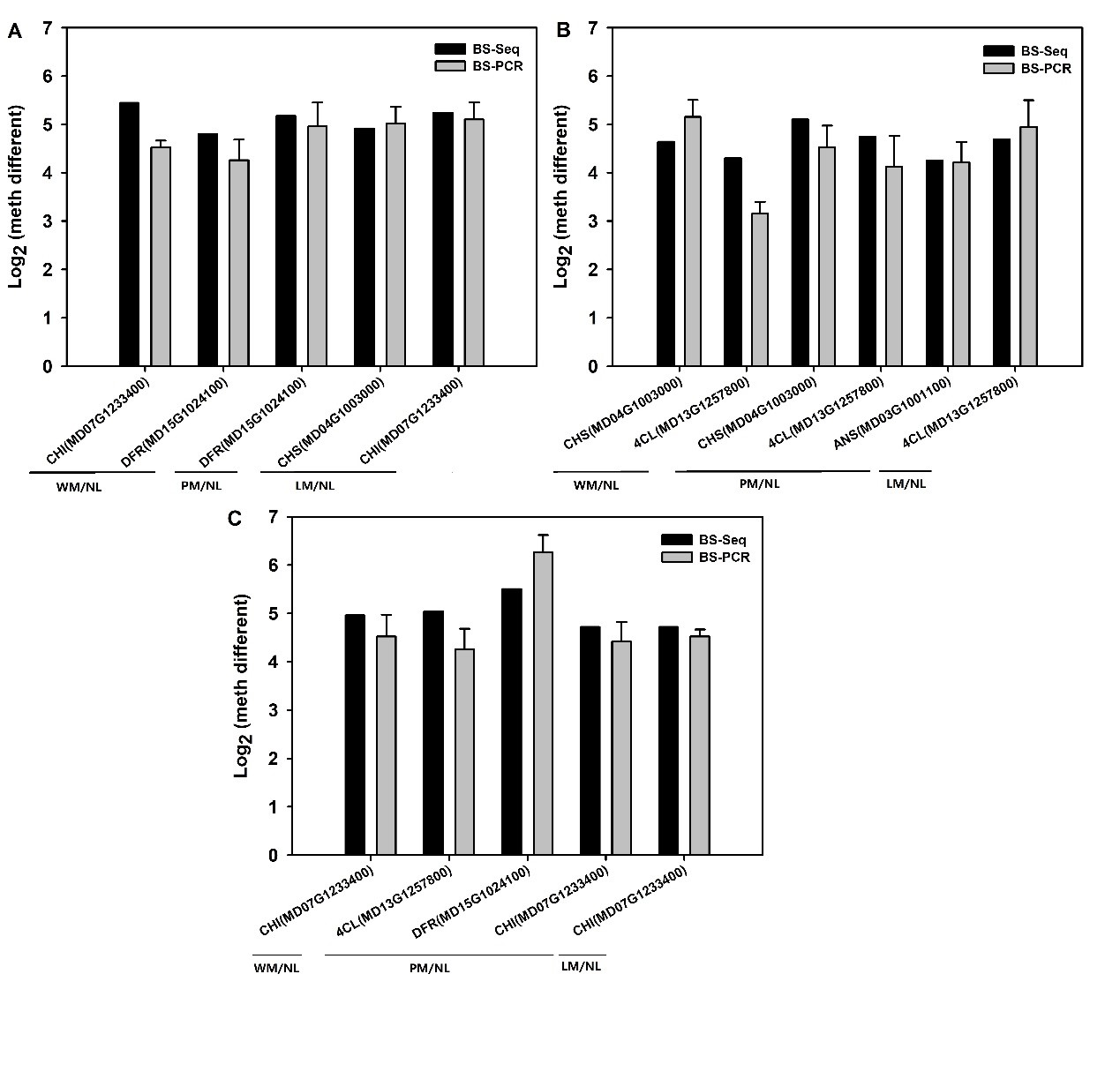


**Fig. S10.** DNA methylation validated with BS-Seq and BS-PCR in WM/NL, PM/NL, and LM/NL. (A) Validation of the DNA methylation of CG. (B) Validation of the DNA methylation of CHG. (C) Validation of the DNA methylation of CHH. The vertical axis represents the value of log2 (meth different) and the horizontal axis indicates candidate genes. The bars represent standard error. Each fragment was subjected to three independent PCRs to produce 12 independent clones for sequencing.


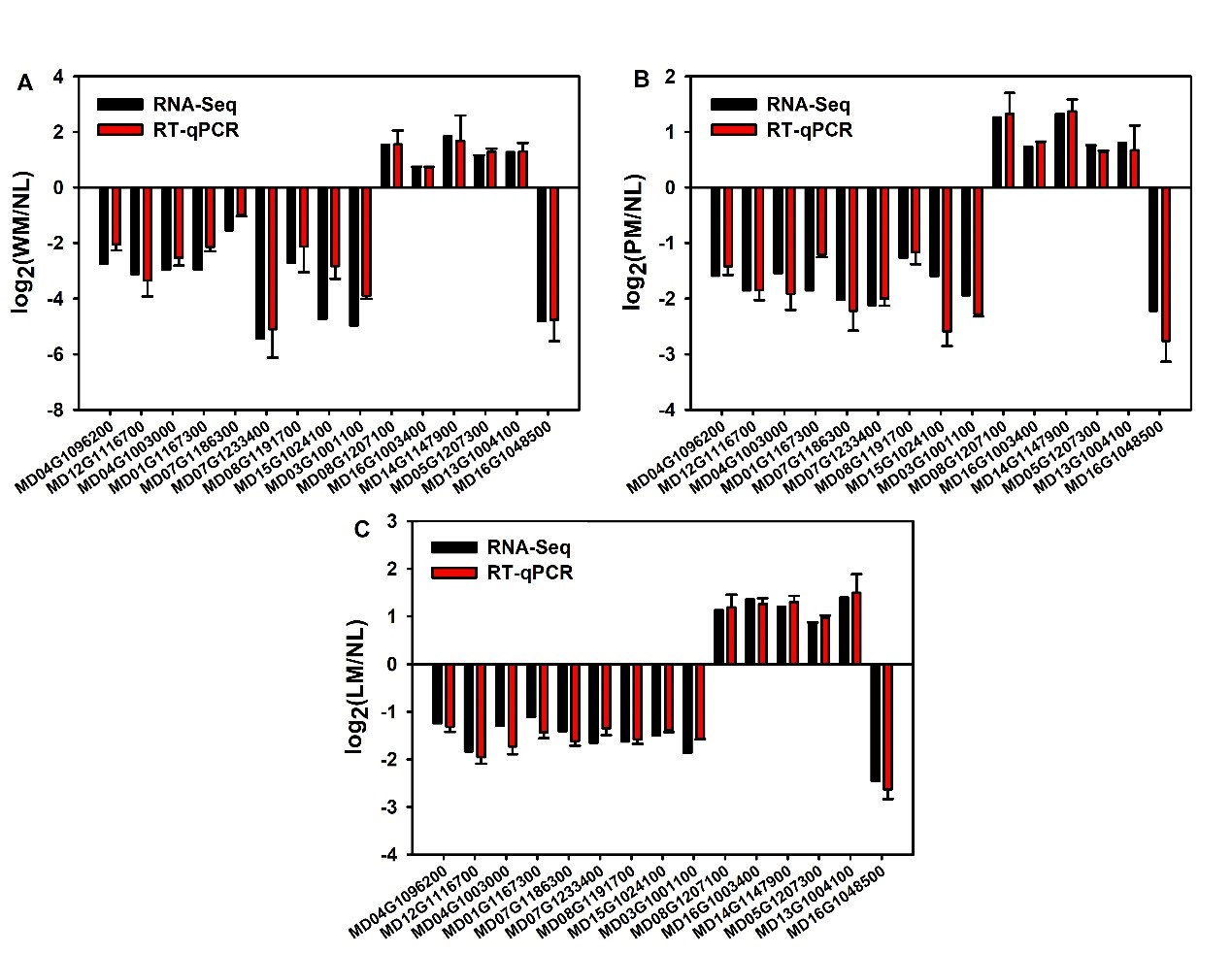


**Fig. S11.** Gene expression validation involved in the chlorophyll, carotenoid and flavonoid biosynthesis pathways. (A) Validation of the expression level of candidate genes in WM/NL. (B) Validation of the expression level of candidate genes in PM/NL. (C) Validation of the expression level of candidate genes in LM/NL. The vertical axis indicates the value of log_2_ (Fold Change) and the horizontal axis represents candidate genes.
